# Unexpected patterns of Epstein–Barr virus transcription revealed by a High throughput PCR array for absolute quantification of viral mRNA

**DOI:** 10.1016/j.virol.2014.10.030

**Published:** 2015-01-01

**Authors:** Rosemary J Tierney, Claire D Shannon-Lowe, Leah Fitzsimmons, Andrew I Bell, Martin Rowe

**Affiliations:** School of Cancer Sciences, College of Medical and Dental Sciences, University of Birmingham, Edgbaston, Birmingham B15 2TT, United Kingdom

**Keywords:** Epstein–Barr virus, HHV4, Herpesvirus, Viral gene expression, Transcription, Lytic replication, Latency, Burkitt lymphoma, Tumor virus

## Abstract

We have validated a flexible, high-throughput and relatively inexpensive RT-QPCR array platform for absolute quantification of Epstein–Barr virus transcripts in different latent and lytic infection states. Several novel observations are reported. First, during infection of normal B cells, Wp-initiated latent gene transcripts remain far more abundant following activation of the Cp promoter than was hitherto suspected. Second, EBNA1 transcript levels are remarkably low in all forms of latency, typically ranging from 1 to 10 transcripts per cell. EBNA3A, -3B and -3C transcripts are likewise very low in Latency III, typically at levels similar to or less than EBNA1 transcripts. Thirdly, a subset of lytic gene transcripts is detectable in Burkitt lymphoma lines at low levels, including: BILF1, which has oncogenic properties, and the poorly characterized LF1, LF2 and LF3 genes. Analysis of seven African BL biopsies confirmed this transcription profile but additionally revealed significant expression of LMP2 transcripts.

## Highlights

• We have validated a QPCR array platform to quantify Epstein–Barr virus transcripts.
• Detection of 45 viral transcripts starting from ng amounts of RNA.
• EBNA1 transcript levels are remarkably low in all forms of latency.
• BILF1, LF1, LF2 and LF3 transcripts are detectable in Burkitt lymphoma lines.
• Significant levels of LMP2 transcripts were also detected in BL biopsies.

## Introduction

Primary infection of resting B cells *in vitro* with EBV leads to the activation of the multimerised Wp promoter and expression of EBNA2, EBNA-LP and latent BHRF1, followed by activation of a second promoter Cp and eventually expression of all six EBNAs (EBNA1, 2, 3A, 3B, 3C and LP) and, from their own promoters, three latent membrane proteins (LMP1, 2A and 2B) (Amoroso et al., 2011, Kelly et al., 2009, Rickinson and Kieff, 2007, Tierney et al., 2011). The non-coding EBERs (Arrand and Rymo, 1982, Lerner et al., 1981), BARTs (Chen et al., 1999, Gilligan et al., 1990, Sadler and Raab-Traub, 1995) and a series of miRNAs (Amoroso et al., 2011, Pfeffer et al., 2004) are also transcribed. This pattern of latent gene expression, which drives B cell growth transformation and the establishment of permanently growing lymphoblastoid cell lines (LCLs), has been classified as Latency III, or Lat III (Rickinson and Kieff, 2007, Rowe et al., 1992).

Alternative patterns of viral gene expression have also been classified. The most restricted, Latency 0 (Lat 0), is the form found in circulating B lymphocytes *in vivo* in healthy virus carriers where all EBV protein expression is silenced, and only the non-coding EBERs, BARTs and miRNAs are transcribed. Latency I (Lat I), identified in Burkitt lymphoma (BL) biopsies and many derived BL cell lines (Rowe et al., 1992), is characterized by a lack of Cp/Wp promoter activity and the expression of a single latent antigen EBNA1 from the alternate Qp promoter (Nonkwelo et al., 1996, Schaefer et al., 1995b), along with expression of the non-coding RNAs. Latency II (Lat II), characteristic of NPC and Hodgkin lymphoma tumor cells, resembles Lat I but with additional expression of LMP1 and LMP2 (Brooks et al., 1992, Deacon et al., 1993). These latency definitions are not absolute, but represent points on a spectrum of EBV gene expression which may occur at different times or different anatomical sites during EBV latency *in vivo* (Thorley-Lawson et al., 2013).

In contrast to the limited number of genes expressed in virus latency, entry into productive lytic cycle results in the temporally co-ordinated expression of over 80 lytic genes (Kieff and Rickinson, 2007). The immediate-early (IE) transactivators, BZLF1 and BRLF1 (Feederle et al., 2000, Takada and Ono, 1989), induce the expression of a number of EBV genes in either a methylation-dependent or methylation-independent manner (Bergbauer et al., 2010, Ramasubramanyan et al., 2012), leading to the expression of early (E) genes, including those required for genome replication, and late (L) genes including structural proteins (Yuan et al., 2006).

We and others have previously reported the quantitation of EBV transcripts in both *in vitro* models and in *ex vivo* samples using reverse transcriptase quantitative PCR (RT-QPCR) (Bell et al., 2006, Dorner et al., 2008, Jochum et al., 2012, Kubota et al., 2008, Kurokawa et al., 2005, Shannon-Lowe et al., 2009, Tierney et al., 2011, Wang et al., 2009, Whitehurst et al., 2013). However these earlier studies only quantified each transcript relative to that in a reference EBV-infected cell line, with a panel of cell lines being used for different latent and lytic transcripts. Due to variations in the efficiency of different PCR reactions and the use of different reference lines, this approach precluded a meaningful comparison of the absolute levels of viral transcripts within a sample. In the present work, we have developed an inexpensive, high throughput method for the absolute quantification of EBV transcripts in small amounts of RNA applicable to a variety of samples including clinical biopsy material. To this end we designed a reference plasmid containing a single copy of 45 different latent and lytic cycle EBV amplicons and 3 cellular control amplicons. Using a 48:48 dynamic array integrated fluidics circuit (IFC) and the Biomark C system (Fluidigm), we were able to simultaneously screen 48 samples with up to 48 different Taqman RT-QPCR assays (Spurgeon et al., 2008). The absolute numbers of each transcript were then determined from standard curves generated from known copy numbers of the reference plasmid. In this paper we have validated this approach and report the quantification of EBV transcripts in different experimental infection models and in clinical Burkitt lymphoma samples. This analysis lead to a number of novel observations that are relevant to a more complete understanding of the transcriptional events following primary infection of B cells and in cell lines established from normal and malignant B cells.

## Results

### Validation of the Fluidigm dynamic array system for EBV transcript detection

**Fig. 1:**
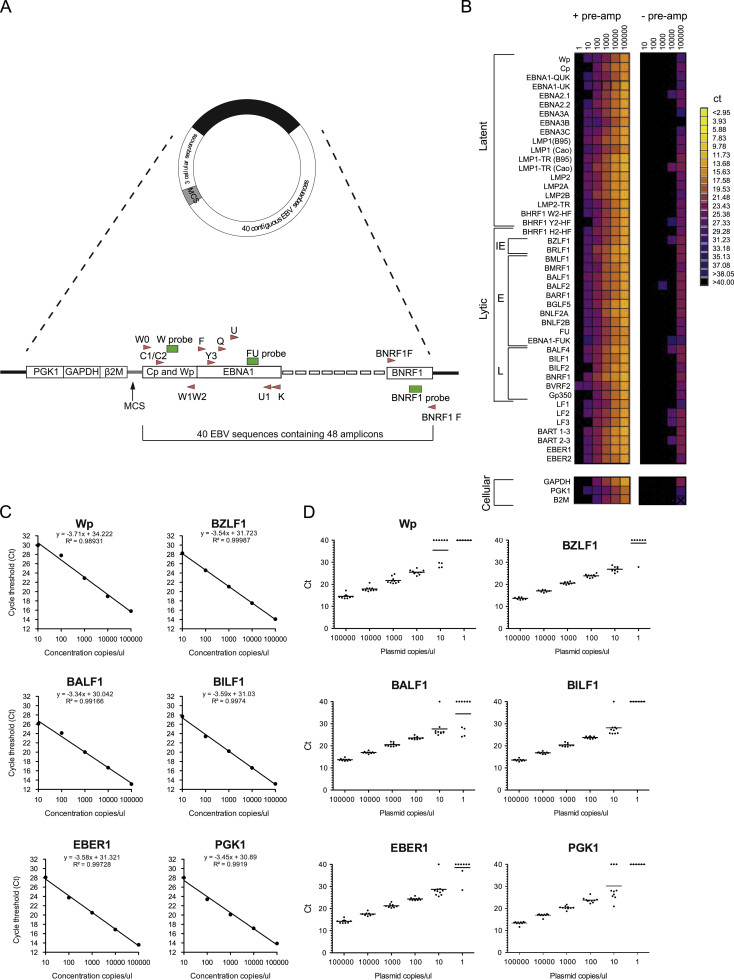
Validation of the 48:48 dynamic array RT-QPCR assays for EBV transcripts. (A) Design of the AQ-plasmid. Further details of the sequences are provided in Supplementary Table S1 and Fig. S1. (B) Heat map summarizing the signals obtained for all 45 PCR assays using 10-fold dilutions of the AQ-plasmids with and without pre-amplification. The scale placed to the right of the heat maps ranges from 3 to 38 *C*_*t*_. (C) Representative standard curves for 5 EBV transcripts and the PGK cellular transcript. Also shown are the best-fit equations which indicate the assays have similar sensitivities while the PCR efficiencies are between 86% and 99%. (D) The reproducibility of the assays for the same 6 representative transcripts is illustrated by the results of 10 independent arrays using independently pre-amplified AQ-plasmid dilutions.

A series of primer and probe combinations were designed for RT-QPCR amplification of 45 EBV amplicons, representing 23 latent cycle transcripts; 2 immediate-early (IE), 11 early (E), 6 late (L) transcripts; 3 poorly characterized RNAs (LF1, LF2 and LF3) and 4 non-coding RNAs (Supplementary Table S1). To determine the absolute levels of each transcript by RT-QPCR, the reference AQ-plasmid was synthesized containing a single copy of each of these amplicons together with sequences recognized by GAPDH, PGK1 and B2-microglobulin Human Endogenous Control TaqMan assays (Life Technologies) (Fig. 1A and Supplementary Fig S1). Following a specific target amplification step, PCR amplification and detection was performed using the Fluidigm Biomark HD and 48:48 dynamic array IFC system (Spurgeon et al., 2008). In a pilot experiment, tenfold dilutions of the AQ-plasmid were subjected to 12 cycles of pre-amplification prior to QPCR with 45 EBV assays and 3 cellular assays, alongside control plasmid dilutions that had not been pre-amplified. Sensitive detection of all the targets was dependent upon the pre-amplification step, as shown by the failure to detect all but the highest concentration of plasmid in the non-amplified controls (Fig. 1B). By contrast, most of the assays using pre-amplified samples were able to detect the 10 copies/µl dilution, with some detecting the 1 copy/µl sample. Fig. 1C shows a panel of 6 representative standard curves drawn from the same data: these include a latent transcript, Wp; an immediate-early (IE) lytic transcript, BZLF1; an early (E) lytic transcript, BALF1; a late (L) lytic transcript, BILF1; a non-coding transcript, EBER1; and a cellular transcript, PGK1. The similarity of the slopes and intercepts indicate comparable sensitivities and PCR amplification efficiencies, while the correlation values show that pre-amplification did not affect the linearity of the reaction. Comparison of the *C*_*t*_ values for the same 6 representative genes obtained from 10 independent experiments demonstrated that the results from the Fluidigm Biomark HD system were reproducible across a 4 to 5 log range (Fig. 1D).

### Absolute quantitation of EBV transcripts following induction of lytic cycle

**Fig. 2:**
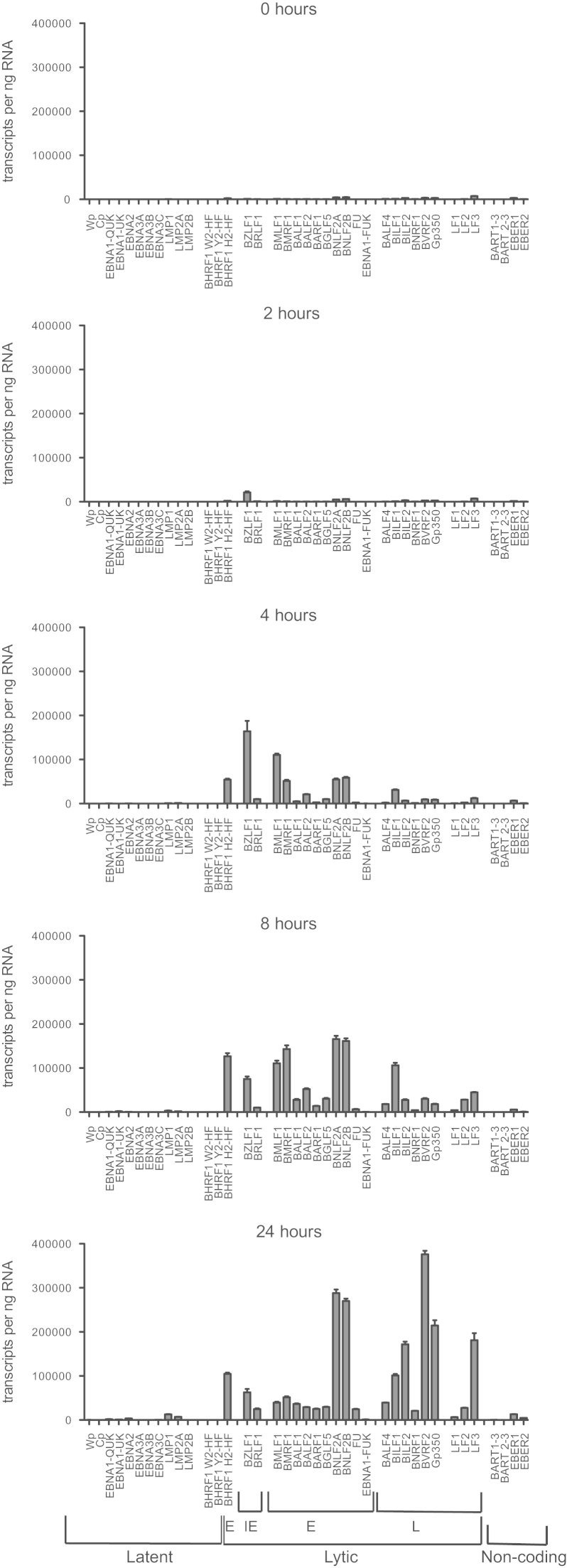
Absolute quantitation of EBV transcripts during induction of lytic cycle. Akata BL cells were induced into lytic cycle by BCR ligation with anti-IgG. Cells were harvested at the indicated time-points and RNA isolated for analysis on the 48:48 dynamic array. Levels of individual latent, immediate early (IE), early (E), late (L) and non-coding transcripts are reported as copies per ng RNA. Data are shown for one representative experiment and the error bars represent the S.E.M of 3 replicate cDNA samples that were independently pre-amplified. Note that BHRF1 can be both a latent transcript (with W2-HF and Y2-HF exon structures transcribed from Cp/Wp) and a lytic transcript (with the H2-HF exon structure transcribed from a separate promoter).

**Fig. 3:**
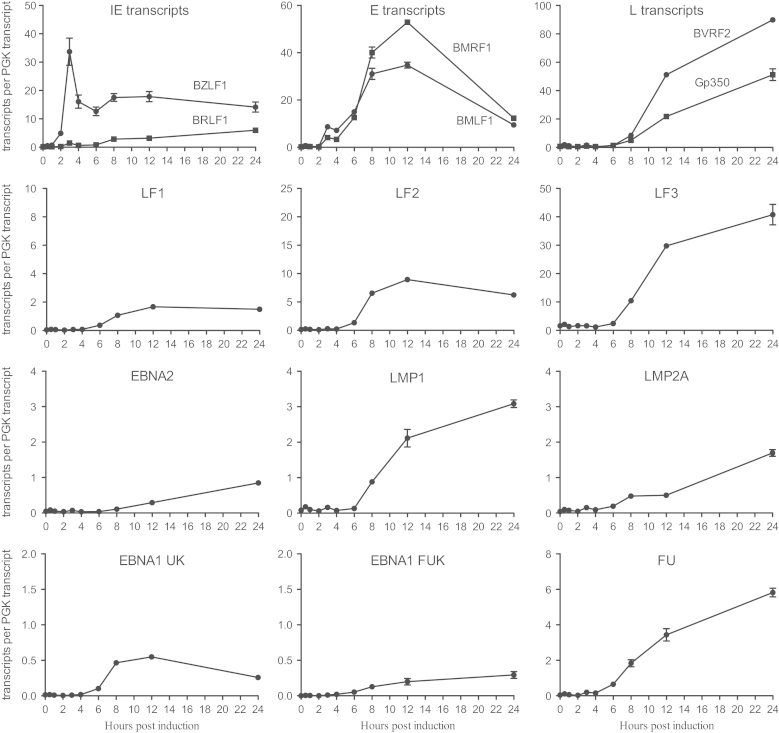
Kinetics of EBV transcript changes during induction of lytic cycle. As in Fig. 2, Akata cells were induced into lytic cycle. Here, the level of EBV transcripts detected in each sample was expressed relative to the number of cellular PGK transcripts detected in each sample. Error bars represent the S.E.M of 3 replicate cDNA samples that were independently pre-amplified.

Having validated the method of high-throughput absolute quantitation using a plasmid control, we next sought to use our panel of EBV assays to characterize the EBV transcriptome following the switch from latency into viral replication. To this end, Akata-BL cells displaying a predominantly Lat I gene expression profile were induced into lytic cycle by B cell receptor cross-linking and cells were harvested at intervals from 0.5 to 72 h for RNA extraction and RT-QPCR analysis. In the representative experiment shown in Fig. 2, 69% of cells had entered the lytic cycle, as judged by flow cytometry of BZLF1-positive cells at 24 h post-induction (data not shown). The RT-QPCR data shown as histograms in Fig. 2 are expressed as the number of transcripts/ng of input RNA. To more readily compare the results between samples taken at different time points, and to correct for possible variations in RNA input, cDNA synthesis and pre-amplification efficiency, these data were also normalized to levels of cellular PGK1 transcripts (Fig. 3 and Supplementary Fig. S2).

Untreated Akata-BL cells (0 h) had very low levels of latent gene transcription and some baseline lytic cycle activity. By 2 h post-induction, there was a clear increase in the number of BZLF transcripts, although interestingly the level of the second IE RNA, BRLF1, was much lower and remained so throughout the time course. BZLF1 was the most abundant transcript at 4 h post-induction when levels peaked around 164,000 transcripts/ng RNA. Several E RNAs were also detectable by this time point, with BHRF1, BMLF1, BMRF1, BNLF2A and BNLF2B being the most abundant. With the exception of BNLF2A and BNLF2B, E gene expression generally continued to rise until 12 h post-induction and then declined. By contrast, L RNAs were much lower at these early times. Consistent with a previous report (Yuan et al., 2006), L gene expression generally peaked at 24 h post-induction although we noted large variations in transcript copy numbers between genes. The LF transcripts are less well characterized than other EBV genes due to their deletion from the B95.8 prototype strain. While levels of LF1 and LF2 peaked at 12 h post induction consistent with E gene expression, LF3 only reached maximal levels at 24 h. Based on these kinetics, LF3 behaves like a L RNA but in fact has previously been defined as an E RNA because its expression is not affected by inhibitors of viral DNA replication (Yuan et al., 2006).

Consistent with previous studies (Amoroso et al., 2011, Rowe et al., 1992, Yuan et al., 2006), increased latent gene transcription also was apparent by 24 h post induction, most notably for LMP1, EBNA2 and LMP2A (Fig. 2, Fig. 3 and Supplementary Fig. S2). However the most abundant of these, LMP1, was still around 30-fold lower than the most abundant lytic transcript BVRF2 (*cf.* 13,000 LMP1 transcripts/ng RNA and 380,000 BVRF2 transcripts/ng RNA). EBNA1 transcripts also increased following lytic cycle induction (as measured by assays detecting QUK-, UK- and FUK-spliced EBNA1 transcripts; Fig. 3 and Supplementary Fig. S2) as a result of activation of the lytic cycle Fp promoter. However, the levels of UK- and FUK-spliced transcripts encoding EBNA1 remained very low and, unexpectedly, represented only about 10% of the total Fp-initiated transcription (FU assay, Fig. 3). Thus our quantitative assays confirm that the majority of FU-spliced transcripts initiating from Fp do not splice into the EBNA1 open reading frame (Schaefer et al., 1995a); where these Fp-initiated transcripts splice to beyond the U exon is presently unknown.

### Absolute quantification of EBV transcripts during primary B cell infection

We next sought to use the expanded panel of PCR assays to more fully characterize and quantify the absolute levels of latent and lytic viral transcripts during EBV-driven transformation of primary resting B cells *in vitro*. CD19-positive B cells were isolated and infected with the 2089 recombinant EBV strain at an MOI of 100, and cells were harvested at multiple time-points for isolation of RNA and quantification of viral gene transcripts.

**Fig. 4:**
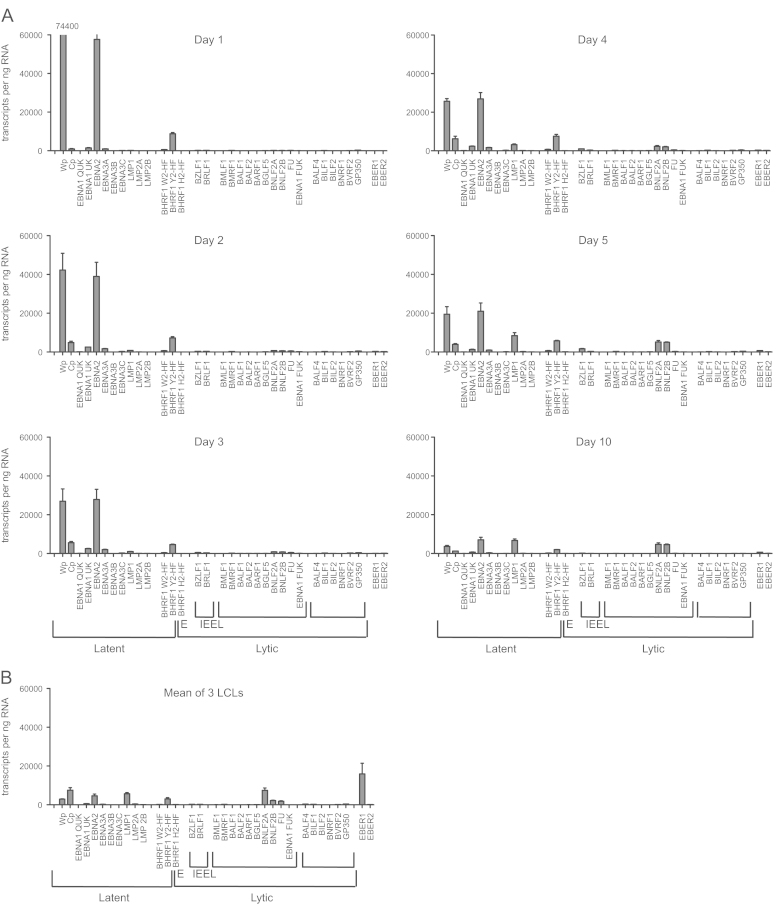
Absolute quantitation of EBV transcripts induced during infection of normal B cells. (A) Primary B cells isolated from peripheral blood were infected at an M.O.I. of 100 with the 2089 recombinant EBV derived from B95.8 strain. Cells were harvested at the indicated time-points and RNA isolated for analysis on the 48:48 dynamic array. Levels of transcripts were measured as number of transcripts per ng RNA, and the data are shown for one representative experiment. Error bars represent the S.E.M of 3 replicate cDNA samples that were independently pre-amplified. (B) For comparison, 3 newly-established LCLs (2–3 months post-infection) were assayed in parallel.

While the kinetics and pattern of latent gene expression largely agreed with published data (Alfieri et al., 1991, Allday et al., 1989, Shannon-Lowe et al., 2005), the absolute numbers of transcripts revealed some unexpected findings (Fig. 4A and Supplementary Fig. S3). At day 1 post infection, the levels of Wp-initiated and EBNA2-specific transcripts already exceeded 60,000 transcripts/ng RNA (Fig. 4A) with lower levels (8700 transcripts/ng RNA) of Y2-HF spliced latent BHRF1 transcripts also present. Notably these initial levels of Wp-initiated EBNA2 and BHRF1 transcripts were around 18-fold higher and 3-fold higher, respectively, than the mean levels in 3 early passage LCLs tested within 3 months post-infection (Fig. 4A and B). Cp-initiated EBNA transcripts together with UK-spliced EBNA1 and EBNA3A transcripts, were also detectable at day 1 post infection but at much lower levels (Fig. 4A and Supplementary Fig. S3).

By day 2 post-infection, Wp activity and EBNA2 transcript levels had started to fall, although they were still by far the most abundant transcripts, while Cp-initiated transcripts increased and LMP1 was now detectable. During the remainder of the time course, Wp activity and EBNA2 and BHRF1 transcript levels continued to fall, while Cp-initiated transcripts remained unchanged. By day 5, LMP1 levels had reached their maximum and EBER1 RNA became detectable. By 10 days post-infection the transcription profile, with the exception of the EBERs, was similar to that of established LCLs (Fig. 4A and B, Supplementary Fig. S3). Surprisingly even after Wp activity had declined at later time points, numbers of Wp-initiated transcripts were still comparable to those of Cp transcripts.

**Fig. 5:**
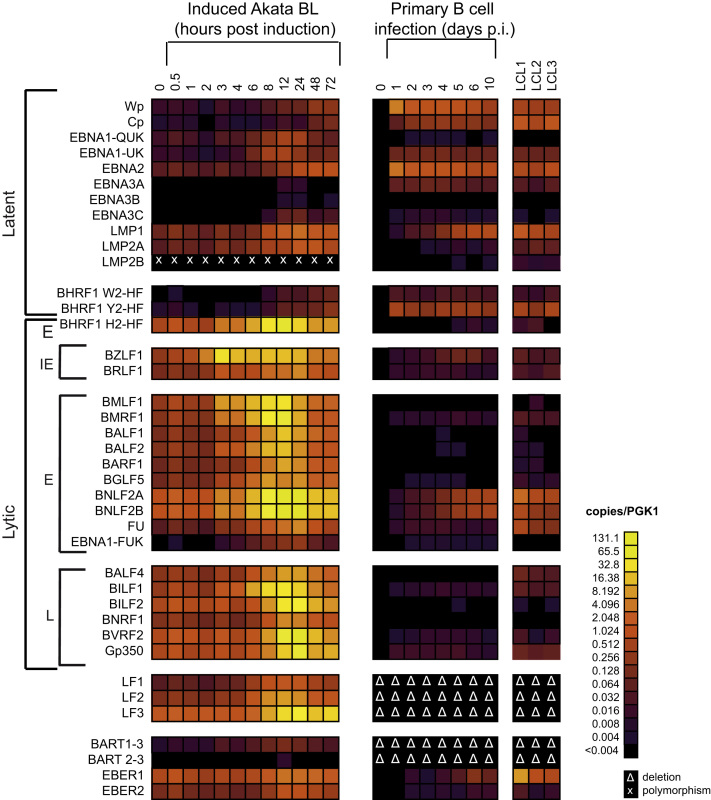
Heat maps to compare EBV transcript levels during lytic cycle induction and infection of primary B cells. Heat map summarizing and comparing the EBV transcription profiles obtained from the experiments described in Fig. 2, Fig. 4. The data are expressed as number of EBV transcripts relative to PGK transcripts, as indicated by the scale to the right of the heat map. Each color change indicates a two-fold increment in transcript levels. Assays affected by polymorphisms or by deletions in the viral genome are indicated by X and ∆, respectively.

In contrast to latent gene transcription, there were very few lytic cycle transcripts during primary infection (Figs. 4A, 5 and Supplementary Fig. S3). Notable exceptions were BNLF2A and BNLF2B which were detected contemporaneously with, and at equivalent levels to, LMP1 (Figs. 4A and 5). However BNLF2A and BNLF2B transcripts are contiguous with the 3′ untranslated region of the LMP1 mRNA and therefore, given the parallels between the BNLF2 and LMP1 signals, it is likely that the BNLF2 signals observed here are predominantly due to LMP1 transcripts. Of the other detectable lytic cycle transcripts, the most abundant was BZLF1 which peaked at 5 days post-infection (Fig. 4A and Supplementary Fig. S3). However this BZLF1 signal was at least 100-fold lower than the levels observed during *bona fide* lytic cycle (*cf.* Figs. 2 and 4A), implying that either less than 0.5% of the cells expressed high levels of BZLF1 or that BZLF1 was uniformly expressed at very low levels in the entire population.

For comparative purposes, the PGK1-normalized changes in EBV gene expression during lytic cycle induction in Akata-BL and during primary B cell infection are shown as heat maps in Fig. 5. Surprisingly the pattern and levels of Lat III-associated latent transcripts expressed at late stages of lytic cycle were similar to those in established LCLs. By contrast the levels of lytic cycle transcripts induced in productively infected Akata-BL cells substantially surpassed any lytic gene expression seen in primary infection or established LCLs. Indeed, the levels of many lytic cycle transcripts exceeded even that of the EBER RNAs which were hitherto regarded as being the most abundant EBV transcripts.

### EBV mRNA within infectious virions

It has been reported that EBV transcripts may be packaged into virus particles and subsequently be transferred to target B cells during primary infection *in vitro* (Jochum et al., 2012). To test this observation, we determined both the number of RNA transcripts and EBV DNA genomes present in virus preparations which had been purified by density gradient centrifugation (Table 1). While many viral transcripts were detectable using our assays, they were present at very low copy numbers with the most abundant being Gp350 RNA (0.007 transcripts/EBV genome). This would suggest that when primary B cells are infected at a multiplicity of infection of 100, on average less than one Gp350 transcript is available for delivery per cell. However this is likely to be a considerable overestimate, as most of the virus remains at the cell surface with the majority of infected cells receiving less than 5 genomes, even at a high multiplicity of infection (Shannon-Lowe et al., 2005). Therefore our findings suggest that incoming virus delivers insignificant amounts of viral transcripts.

**Table 1:** EBV mRNAs detected in mature infectious virions.

Transcript	Number of transcripts per µl RNA^a^	Number of transcripts per virion b
Wp	38	<
Cp	5	<
QUK	0	<
UK	47	<
FU	28	<
FUK	0	<
EBNA2	1,381	<
EBNA3A	9,240	0.003
EBNA3B	5,791	0.002
EBNA3C	305	<
LMP1	0	<
LMP2A	10,280	0.004
LMP2B	5,717	0.002
W2-HF	0	<
Y2-HF	0	<
H2-HF	292	<
BZLF1	10,594	0.004
BRLF1	6,325	0.002
BMLF1	14	<
BMRF1	2,548	0.001
BALF1	5,099	0.002
BALF2	3,187	0.001
BARF1	5,547	0.002
BGLF5	3,475	0.001
BNLF2A	11,064	0.004
BNLF2B	7,826	0.003
BALF4	5,261	0.002
BILF1	6,077	0.002
BILF2	5,530	0.002
BNRF1	4,354	0.002
BVRF2	6,626	0.002
Gp350	19,150	0.007
EBER1	9,013	0.003
EBER2	7,058	0.003

a Corresponds to input from 2.7×10^6^ virions.

b < Indicates values less than 0.001 transcripts/virus genome.

### Quantitation of virus transcripts in different forms of EBV latency

**Fig. 6:**
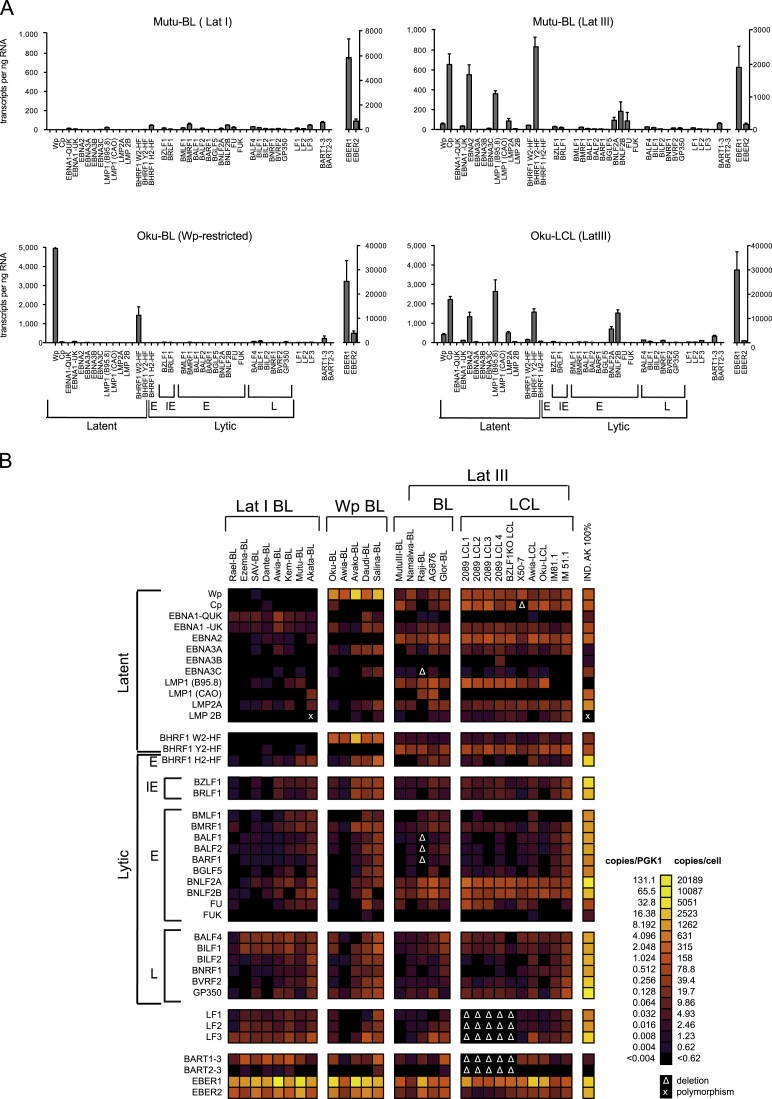
Quantitation of EBV transcripts in B cell lines displaying different forms of latency. (A) Array data obtained from 3 representative BL lines displaying different forms of latency and from an LCL established by infection of normal B cells with the strain of EBV isolated from Oku BL. Results are expressed as number of EBV transcripts per ng RNA. (B) Heat map of data obtained from 8 Lat I BL lines, 5 Wp BL lines, 5 Lat III BL lines, and 10 LCLs. For comparison, results for sorted 100% lytically infected AKBM cells are also shown in the rightmost column. The heat maps were generated exactly as in Fig. 5.

We next compared the absolute levels of EBV transcripts in a panel of 8 Lat I BL lines, 5 Wp-restricted BL lines, 5 Lat III BL lines and 10 Lat III LCLs; note many of these cell lines had previously been tested by relative gene expression studies using a limited number of assays (Amoroso et al., 2011). Fig. 6A shows the absolute numbers of each transcript expressed as copies/ng RNA from four representative cell lines, while Fig. 6B shows PGK1-normalized data from all 28 lines in the form of a heat map. Mutu I BL and Mutu III BL are well characterized sub-clones derived from the same parental biopsy sample but with Lat I and Lat III patterns of gene expression, respectively (Gregory et al., 1990). Oku-BL is a Wp-restricted BL line containing both a transcriptionally active EBNA2-deleted EBV genome and a silent wild-type genome (Kelly et al., 2002), while Oku-LCL was derived by *in vitro* transformation of normal B cells with the wild-type Oku virus.

As expected, the non-coding EBERs were the most abundant EBV transcript in all cell lines regardless of latency state, and these data are therefore displayed on separate axes. Although there was a large variation in the levels of EBER transcripts (63–66,532 transcripts/ng RNA), EBER1 levels were consistently higher (median, 11,773) than EBER2 (median 946). The non-coding BART RNAs were also ubiquitously expressed with the BART exon 1–3 spliced transcript always more abundant (median, 164; range, 2–2535 transcripts/ng RNA) than the exon 2–3 spliced form which was barely detectable (median, 2; range, 0.0004–47 transcripts/ng RNA), a result that had been masked in previous studies (Amoroso et al., 2011) using relative measurements.

Analysis of Lat I BL lines, exemplified by Mutu-I BL (Fig. 6A and B), confirmed that QUK-spliced EBNA1 transcripts are the most common antigen-coding latent transcripts in this form of latency (median 43 transcript/ng; range 14–202). Many lytic cycle RNAs were also present at levels comparable to EBNA1, which we presume are generated by a minor subpopulation of lytically infected cells. However several lytic RNAs were consistently detected at levels in excess of that which might be explained by spontaneous reactivation; these included LF3 (median 195 transcripts/ng; range 48–1370), BALF4 (median 171 transcripts/ng; range 8–558) and BILF1 (median 168 transcripts/ng; range 14–433), (Fig. 6B and Supplementary Fig. S4).

Wp-restricted BL lines are characterized by the presence of an EBNA2-deleted genome and the expression of EBNA-LP, BHRF1, EBNA1 and the EBNA3 family of genes, all driven from Wp, and expression of the EBER and BART RNAs (Kelly et al., 2006, Kelly et al., 2009). In the representative Wp-restricted Oku-BL cell line (Fig. 6A) there were almost 5000 Wp-initiated transcripts/ng RNA; W2-HF spliced BHRF1 transcripts were the most abundant component of these Wp-initiated transcripts. A similar situation was found in the other Wp-restricted BL lines, as clearly shown on the heat map (Fig. 6B). Only the EBER RNAs ever reached such consistently high levels of transcription. In contrast the other transcripts present in Wp-restricted latency, EBNA1, EBNA3A, EBNA3B, and EBNA3C, were expressed at much lower levels.

Lat III lines are characterized by the transcription of all six EBNAs (either from Cp and/or Wp), LMPs, BARTs and EBERs. The representative Lat III lines, Mutu-III BL and Oku-LCL (Fig. 6A), show a dominance of Cp-initiated transcripts over Wp-initiated transcripts that was a characteristic of most Lat III lines; a notable exception in our series was the X50-7 LCL, which is unusual in having a genomic deletion encompassing Cp (Yandava and Speck, 1992). In all Lat III lines, EBNA2 and latent BHRF1 (Y2-HF splice) transcripts were the principal products of the Cp/Wp promoters. As noted in the other forms of latency, relatively few EBNA1 and EBNA3A, -3B and -3C transcripts were detected, whereas LMP1 and LMP2 transcripts were more abundant and were often detected at levels comparable to EBNA2 transcripts.

### Verifying the low number of EBNA1 transcripts per cell

Whilst EBNA1 transcripts were readily detected in all EBV-positive cell lines, our data suggest that there might be as little as 1 transcript per cell in some cases, assuming that a typical cell contains about 30–35 pg RNA. To investigate this more accurately, we sorted between 1 and 100 cells directly into replicate wells of a PCR plate and then subjected the samples to pre-amplification and RT-QPCR without prior isolation of RNA. Using linear regression analyses of data from three LCLs and two Lat I BL lines, the mean number of UK-spliced EBNA1 transcripts was 7.0±1.5 transcripts per cell (Supplementary Table S2). In the same experiments, we observed an average of 154±30 cellular PGK transcripts per cell. As the level of PGK transcripts appears relatively constant between different lines and different EBV latencies, this result was used to estimate the approximate number of all viral transcripts per cell from the previously determined ratio of EBV transcripts to PGK transcripts; this conversion has been applied to the scale of the heat maps shown in Fig. 6B and in Supplementary Table S3.

### Quantifying EBV transcripts in Burkitt lymphoma biopsies

**Fig. 7:**
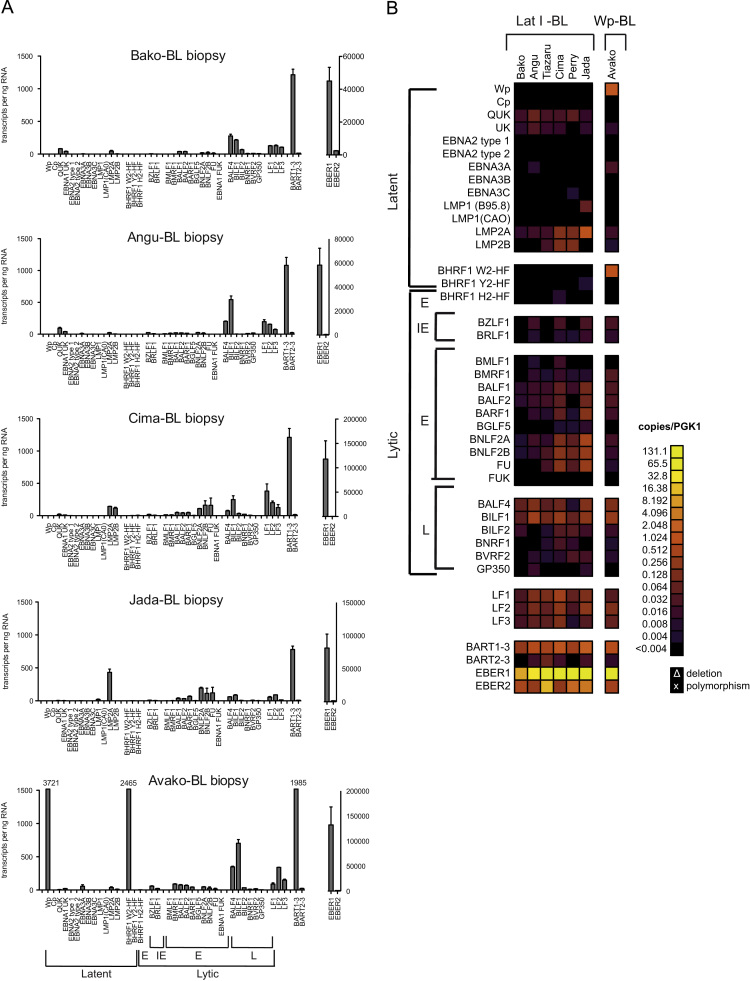
Quantitation of EBV transcripts in BL biopsy samples. (A) Array data obtained from 5 BL biopsy samples; results are expressed as number of EBV transcripts per ng RNA. (B) Heat map summary of array data obtained from all 7 BL biopsies.

One feature of the Dynamic Array RT-QPCR method is that only a very small volume of sample is required to generate quantitative transcription data for 48 individual QPCR assays. This allowed us to examine a series of seven African BL biopsy samples for which very limited amounts of material were available. The data for 5 samples, expressed as transcripts/ng of input RNA, are shown on histograms in Fig. 7A and the PGK1-normalized data for all seven samples are shown in the heat maps in Fig. 7B.

Three observations can be made from these biopsy data. First, the gene expression patterns in the BL biopsies are broadly consistent with those observed with derived BL lines (*cf* Figs. 6B and 7B), with the Wp, BHRF1 and QUK expression profiles readily distinguishing between Lat I and Wp-restricted forms of BL. Second, the striking similarity between the PGK1-normalized data for BL lines and biopsies included the detection of BALF4, BILF1 and LF1–3 transcripts at levels that were consistently higher than the other lytic cycle transcripts. Third, in contrast to most BL lines, LMP2 expression was readily detectable in all seven biopsies, in some cases at levels comparable to those seen in LCLs (*cf.* Figs. 6B and 7B). LMP2A transcripts have previously been observed in some BL biopsies using non-quantitative end-point PCR assays (Tao et al., 1998), but our data suggest that their expression may be a more consistent feature of BL tumors than is apparent in derived BL lines.

## Discussion

In this study, we have optimized and validated the high throughput Fluidigm Dynamic Array system to simultaneously quantify 48 EBV and cellular transcripts in up to 48 RNA samples. This platform is flexible such that individual QPCR assays can easily be substituted according to the needs of a particular experiment and expanded to a 96×96 format if screening for additional transcripts is required in the future. While PCR-based array profiling of KSHV and EBV transcripts in primary Kaposi sarcoma lesions and virus-positive cell lines has been reported previously (Dittmer, 2003, Fakhari and Dittmer, 2002, Kurokawa et al., 2005, Wang et al., 2009, Whitehurst et al., 2013), the present work provides two novel features. First, the Fluidigm system requires very little starting material (less than 25 ng RNA) making it suitable for analyzing small clinical samples. Second, we have developed a common standard that allows absolute quantification of transcript numbers rather than measuring EBV transcript levels relative to their expression in a panel of reference cell lines.

Due to developments in sequencing technologies, RNA-Seq is rapidly becoming the gold standard method for gene expression profiling, and two recent RNA-Seq studies have characterized in detail the EBV transcriptome in Lat I BL cells (Concha et al., 2012, Lin et al., 2010). In contrast to our QPCR approach, RNA-Seq can identify both previously characterized EBV transcripts and novel viral transcripts. However for most laboratories RNA-Seq remains prohibitively expensive for screening EBV transcription in large panels of samples. Another limitation of RNA-Seq is that transcripts derived from repetitive sequences (which constitute as much as 20% of the EBV genome) cannot be reliably mapped and quantified; these include the 5′ regions of the Cp/Wp-initiated transcripts and LF3 transcripts. The Dynamic Array platform used in the present study is not intended to compete with RNA-Seq, rather it is a complementary approach that represents a relatively low-cost option for high-throughput, sensitive, and reliable simultaneous assay of multiple EBV transcripts from small amounts of material.

One striking observation from our analyses of EBV transcription was the low levels of EBNA1 transcripts. Typically, there appear to be fewer than 10 EBNA1 transcripts per cell (and some cases only 1 transcript per cell) both in Cp/Wp-using virus-transformed B cells and Qp-using malignant BL tumor cells. Actually, this is in agreement with previously reported RNA-Seq data (Lin et al., 2010) obtained from two representative Lat I BL lines, Mutu-I BL and Akata BL, both of which are included in our present study. EBNA1 transcription has been reported to occur transiently during cell division (Chau et al., 2008, Davenport and Pagano, 1999), which may in part explain the very low average steady state levels of transcripts. Nevertheless, EBNA1 protein is detectable at constant levels at all stages of the cell cycle due to its very long half life, which can be in excess of 48 h (Davenport and Pagano, 1999). Interestingly, the EBNA3 family transcripts, which are expressed during primary infection of B cells and in lines displaying Latency III or Wp-restricted latency, are also present at levels similar to or less abundant than the EBNA1 transcripts. Again, the EBNA3 proteins are very stable and have slow turnover rates (Touitou et al., 2005). Notably, a global quantitation of cellular mRNA and protein copies/cell (Schwanhausser et al., 2011) suggests that genes with steady state levels of less than 10 transcripts/cell may still produce between 10^2^ and 10^7^ steady state copies of protein. Moreover there does not seem to be a simple correlation between the number of mRNA and protein molecules, and neither does the protein half-life necessarily predict how many copies of protein might be produced from a small number of mRNA copies; an extreme example is Histone H1.2, which has an average of 4.3 copies of mRNA and 1.8×10^7^ copies of protein/cell (Supplementary Table S3).

Our assays of primary B cell infection confirmed the well established time course of Wp-initiated EBNA2 and BHRF1 transcription, followed by activation of Cp and transcription of the remaining EBNAs and LMPs (Alfieri et al., 1991, Shannon-Lowe et al., 2005, Woisetschlaeger et al., 1990). In contrast to an earlier study (Jochum et al., 2012), our data (Table 1) suggest that virion-delivered EBV RNAs do not significantly contribute to the levels of these initial transcripts. We speculate that these contradictory findings may be explained by exosome contamination in unpurified virus preparations used in the previous study, since it is reported that exosomes secreted from EBV-positive B cells contain EBV-encoded RNAs, including EBERs and miRNAs (Ahmed et al., 2014, Pegtel et al., 2010).

A second unexpected observation from our quantitative analyses of primary B cell infection concerned the levels of Cp/Wp-derived transcripts. Wp-initiated transcripts and EBNA2 encoding transcripts were remarkably high at day 1 post infection, considerably higher than found in established LCLs, which is consistent with the activation of multiple copies of Wp driving their transcription (Tierney et al., 2011). The subsequent switch to EBNA transcription from the single copy Cp promoter results in the lower stable levels of EBNA2 transcripts in the outgrowing LCLs. Although Cp-initiated transcription increases temporally as Wp-initiated transcription wanes, what was not appreciated from previous studies was that even when the levels of Wp transcripts have declined to the minimal levels observed in LCLs, their number is still comparable to, and sometimes exceeds, the number of Cp-derived transcripts. This observation necessitates a rethink of the relative importance of Cp and Wp promoter usage in establishing persistent latent infection.

Our data also allow us to comment on the expression of lytic cycle genes during EBV transformation of B cells. Although virion-delivered transcripts may be negligible (Table 1), it is clear that lytic cycle transcription does occur following primary infection of B cells (Kalla et al., 2010, Shannon-Lowe et al., 2009, Wen et al., 2007). Consistent with previous reports, BZLF1 was the most abundant lytic cycle gene with a burst of transcription starting between day 4 and day 6 post infection. However the PGK1-normalized BZLF1 transcript level at day 5 post-infection was 300-fold less than that of productively-infected Akata-BL cells; at present we cannot distinguish whether these signals are derived from a very small number of infected cells undergoing spontaneous reactivation or a general low level of BZLF1 transcription in all the culture. However the lower amounts of other lytic cycle transcripts detected during B cell transformation would indicate that the cells are not in full productive cycle at this stage, consistent with published data that infectious virus does not become detectable until after 13 days post-infection (Kalla et al., 2010). The failure to initiate full lytic cycle immediately post-infection is likely a result of the lack of CpG methylation on the incoming EBV genome, as binding of the BZLF1 transactivator to several lytic cycle promoters is dependent on DNA methylation (Bergbauer et al., 2010, Kalla et al., 2010, Kalla et al., 2012).

Interestingly, our analyses indicated that certain lytic cycle genes were in fact more widely expressed than previously recognized during latency (Supplementary Figs. S3 and S4). These included the vBcl2 homologue BHRF1 which is expressed at low levels during primary infection of B cells and in Latency III LCLs, and at much higher levels in the Wp-restricted subset of BLs carrying an EBNA2 gene-deleted EBV genome (Kelly et al., 2009). BHRF1 has been postulated to act together with BALF1 to protect B cells from apoptosis during primary infection (Altmann and Hammerschmidt, 2005, Kalla and Hammerschmidt, 2012). In our hands, however, whilst BHRF1 transcripts were readily detected as early as day 1, BALF1 transcripts were undetectable at this time point and, when detected at later time-points, were typically at around 100-fold lower amounts than BHRF1.

We also detected several other lytic cycle genes in one or more types of latency at levels that could not be accounted for by contamination with a minor subpopulation of productively infected cells; these included LF3, BALF4, BILF1 and, to a lesser extent, LF1 and LF2 (Supplementary Fig. S4). These genes appeared to be expressed even during Lat I infection in BL lines. Using RNA-Seq, Lin et al. reported that LF3 was the most abundant EBV transcript in Lat I BL lines, at levels higher than 98% of cellular transcripts (Lin et al., 2010). Although we found LF3 to be robustly expressed in Lat I BL, we did not observe such high levels as described by Lin et al. This discrepancy is likely due to LF3 being partially encoded by the IR4 repeat; while our PCR assay was designed to detect the unique region of the LF3 transcript, RNA-Seq reads of the IR4 repeat sequences may greatly overestimate the frequency of LF3 transcripts. The biological significance of LF3 and BALF4 expression in latent infection are still unclear. On the other hand, the expression of BILF1 in latency is intriguing as this gene encodes a constitutively active vGPCR with oncogenic potential (Beisser et al., 2005, Lyngaa et al., 2010, Paulsen et al., 2005).

This study also validates the applicability of our high-throughput assays for analyzing EBV gene expression in small amounts of RNA isolated from *ex vivo* tumor biopsies. Similar to BL cell lines, the biopsy samples consistently contained higher than expected amounts of LF1, LF2, LF3, BILF1 and BALF4 transcripts. In addition, LMP2A and, in some cases, LMP2B transcripts were expressed in BL biopsies at significant levels in the absence of Cp/Wp driven EBNA, or LMP1 transcripts. The detection of LMP2 RNA in BL biopsies has been previously reported (Bell et al., 2006, Niedobitek et al., 1995, Xue et al., 2002), although LMP2 protein expression has not been reported, possibly due to a relatively poor affinity of the available antibodies. Given the known functions of LMP2A, which include signaling through the BCR pathway and modulation of cell death and proliferation in experimental models (Caldwell et al., 1998, Mancao and Hammerschmidt, 2007), its expression in BL would be consistent with the properties of this tumor.

The results with the panel of BL biopsy derived RNA samples, including one sample from a Wp-restricted tumor, Avako, is of particular interest to us as it confirms that our method has the potential to be applied to high-throughput screening of clinical material to identify the true frequency of Wp-restricted BL tumors and to correlate the results with clinical outcome. A remarkable consequence of Wp-restricted latency in BL lines is that it confers a substantial anti-apoptotic phenotype relative to Latency I BL lines (Kelly et al., 2005). It is reasonable to presume, but has not yet been tested, that Wp-restricted and Latency I BL tumors might differ in clinical presentation and responses to therapy.

## Materials and methods

### Cell culture

The cell lines used in this work were: Latency I (Lat I) Burkitt lymphoma-derived cell lines Rael-BL, Ezema-BL, Sav-BL, Dante-BL, Awia-BL, Kem-BL, MutuI-BL and Akata-BL; the Wp-restricted BL lines Oku-BL, Awia-BL, Avako-BL, Daudi-BL and Salina-BL; the Lat III BL lines MutuIII-BL, Namalwa-BL, Raji-BL, Ag876 and Glor-BL; the long term Lat III virus-transformed 2089 LCL, BZLF1KO LCL, X50-7 LCL, Oku-LCL, Awia-LCL, IM51.1 LCL and IM 81.1 LCL and three recently established LCLs generated using 2089 virus (Amoroso et al., 2011, Kelly et al., 2005). Two other lines derived from Akata-BL were also used: Akata-GFP-BL is derived from the re-infection of EBV-loss Akata-BL cells with a recombinant Akata virus constitutively expressing green fluorescent protein (GFP) (Kanda et al., 2004) while AKBM cells contain a stably transfected GFP reporter under the control of the early BMRF1 promoter (Ressing et al., 2005). Cell lines were maintained in exponential growth in RPMI supplemented with 10% fetal calf serum and 2 mM glutamine at 37 °C in 5% CO_2_.

### Biopsy samples

RNA was obtained from a series of previously-described endemic BL tumor biopsies obtained from the West Nile region of Uganda (Bell et al., 2006, Habeshaw et al., 1999). The use of patient samples was approved by The West Midlands and The Black Country Committee of the National Research Ethics Service, UK (REC reference 14/WM/0001).

### Reactivation of EBV in Akata cells and isolation of cells in the lytic cycle

Akata-GFP-BL cells were induced into lytic cycle by crosslinking surface immunoglobulin with goat anti-human IgG antibody (Ressing et al., 2005). Aliquots of 3×10^6^ induced cells were harvested at the indicated time points after the initial addition of antibody. At 24 h post induction an aliquot of cells was also subjected to staining with BZLF1-specific antibody and analyzed by flow cytometry to determine the percentage of the cell population that had entered into lytic cycle. To purify productively infected cells, AKBM cells were induced into lytic cells by IgG cross-linking and GFP-positive cells were isolated using a MoFlo cell sorter (Beckman Coulter).

### Primary B cell infection

B cells were isolated from apheresis cones (NHS Blood and Transplant) by positive selection with CD19 Dynabeads (Life Technologies) followed by removal of the beads with Detachabead (Life Technologies). B cells were then infected with recombinant 2089 EBV at 100 MOI as previously described (Shannon-Lowe et al., 2005) and cells harvested at specific time points, washed, pelleted and frozen at −80 °C.

### Preparation of EBV virions

Virus preparations were made from 293 cells containing the 2089 EBV recombinant virus co-transfected with BZLF1 and BALF4 expression plasmids (Shannon-Lowe et al., 2005). Supernatant medium was harvested after 3 days, passed through a 0.8 µm filter and the virus particles purified by density gradient centrifugation through Optiprep medium (Axis-Shield). Virion concentration was determined by real-time Taqman PCR for the EBV Pol gene, as previously described (Shannon-Lowe et al., 2005). For RNA isolation, the sample was pre-treated with RNAse and DNAse I to remove any non-encapsidated nucleic acids prior to RNA extraction from the virion particles.

### RNA extraction and cDNA synthesis

Aliquots of up to 1 µg RNA, prepared using a Nucleospin II kit (Macherey-Nagel), were subjected to an additional DNAse I treatment using a DNA-free kit (Life Technologies) according to the manufacturer’s instructions. cDNA was made from up to 1 µg RNA using QScript (VWR) according to the manufacturer’s protocol and generally diluted to 20 ng/µl.

### Plasmid for absolute quantitation (AQ)

A 4951 base pair DNA sequence was designed containing 45 contiguous latent and lytic EBV amplicons and 3 cellular amplicons (Supplementary Fig. S1). In cases where the primer-probe combination amplified a single exon, the native EBV sequence was used as the target amplicon. In cases of alternatively spliced viral transcripts which shared one or more common exons (W0-W1-W2 and C1-C2-W1-W2 spliced Wp/Cp transcripts, FQ-U-K/Q-U-K/U-K spliced EBNA1 RNA, W2-HF/Y2HF/H2-HF BHRF1 RNA and exon 1–3/exon 2–3 spliced BART RNA), composite target sequences were designed containing the appropriate primer-probe binding sites and which generated PCR products of a similar size to the native EBV transcript (Fig. 1A and Supplementary Fig. S1). In addition, for EBNA2 and LMP1, two alternative amplicons representing common EBV sequence variants were included. This sequence was commercially synthesized and inserted into the pUC57 vector (GenScript) and termed the AQ-plasmid. Serial dilutions of the AQ-plasmid in the range 10^−1^–10^5^ copies/µl were subsequently used to generate QPCR standard curves.

### Design of TaqMan real-time PCR assays

Forty five primer-probe combinations to detect selected EBV transcripts were designed using Primer Express (Life Technologies) under the default parameter settings. All primer-probe sequences (detailed in Supplementary Table S1) were based on the B95-8 reference genome (de Jesus et al., 2003) and were chosen to avoid reported EBV sequence variations. In the case of assays which detect a single exon, the assay name refers to the gene location of the primer-probe combination. Primer-probe combinations to detect alternatively spliced transcripts spanned a splice junction and are referred to both by the gene name and exon structure. Several of these assays have been described previously (Amoroso et al., 2011, Bell et al., 2006, Croft et al., 2009, Kelly et al., 2009). Note that due to a number of overlapping transcription units in the EBV genome, several assays may potentially detect more than one transcript. These are BNLF2A and BNLF2B, which may also detect the 3′ UTR of LMP1 transcripts; BMRF1 which may also detect overlapping BaRF1 transcripts and BNRF1 which may detect the 3′ UTR of LMP2 transcripts. Primers were obtained from Alta Bioscience, University of Birmingham and FAM-TAMRA labeled TaqMan probes were obtained from Eurogentec. Endogenous control assays for Glyceraldehyde 3-phosphate dehydrogenase (GAPDH, assay ID hs99999905.m1 FAM/TAMRA), Beta-2-microglobulin (B2M, assay ID hs00187842 FAM/TAMRA) and Phosphoglycerate kinase 1 (PGK1, assay ID hs99999906.m1 FAM/TAMRA) were obtained from Life Technologies.

### Specific target amplification and 48:48 dynamic array IFC analysis

Specific target amplification (STA) was carried out on 1.25µl (25 ng) of DNAse I-treated RNA with 2.5 µl of 2× TaqMan PreAmp Master Mix (Life Technologies) and 1.25 µl 0.2× primer mix (45 EBV and 3 cellular 20× TaqMan assays diluted in DNA Suspension Buffer (10 mM Tris, pH 8.0, 0.1 mM EDTA). Reactions were subjected to 95 °C for 10 min, followed by 12 cycles of 95 °C for 15 s and 60 °C for 4 min. These pre-amplified samples were then diluted 1:5 with DNA Suspension Buffer prior to array analysis, except for virion samples which were not diluted. For absolute quantification 1.25 µl aliquots of ten-fold dilutions of AQ-plasmid standards (10^5^–10^−1^ copies/µl) were also subjected to the same pre-amplification and dilution steps. The 48:48 dynamic array IFC was prepared and run in a Biomark HD instrument according to manufacturer’s instructions using the standard v1 protocol, and the data were analyzed using Biomark Real-Time PCR Analysis Software Version 2.0 (Fluidigm).
