# Supplementary material for: Unexpected patterns of Epstein–Barr virus transcription revealed by a High throughput PCR array for absolute quantification of viral mRNA
Source: Virology. 2015 Jan 1;474:117–30. doi: 10.1016/j.virol.2014.10.030 (PMC4266535; doi:10.1016/j.virol.2014.10.030)
Supplement: Supplementary file 1 — Supplementary material [file mmc1.pdf]

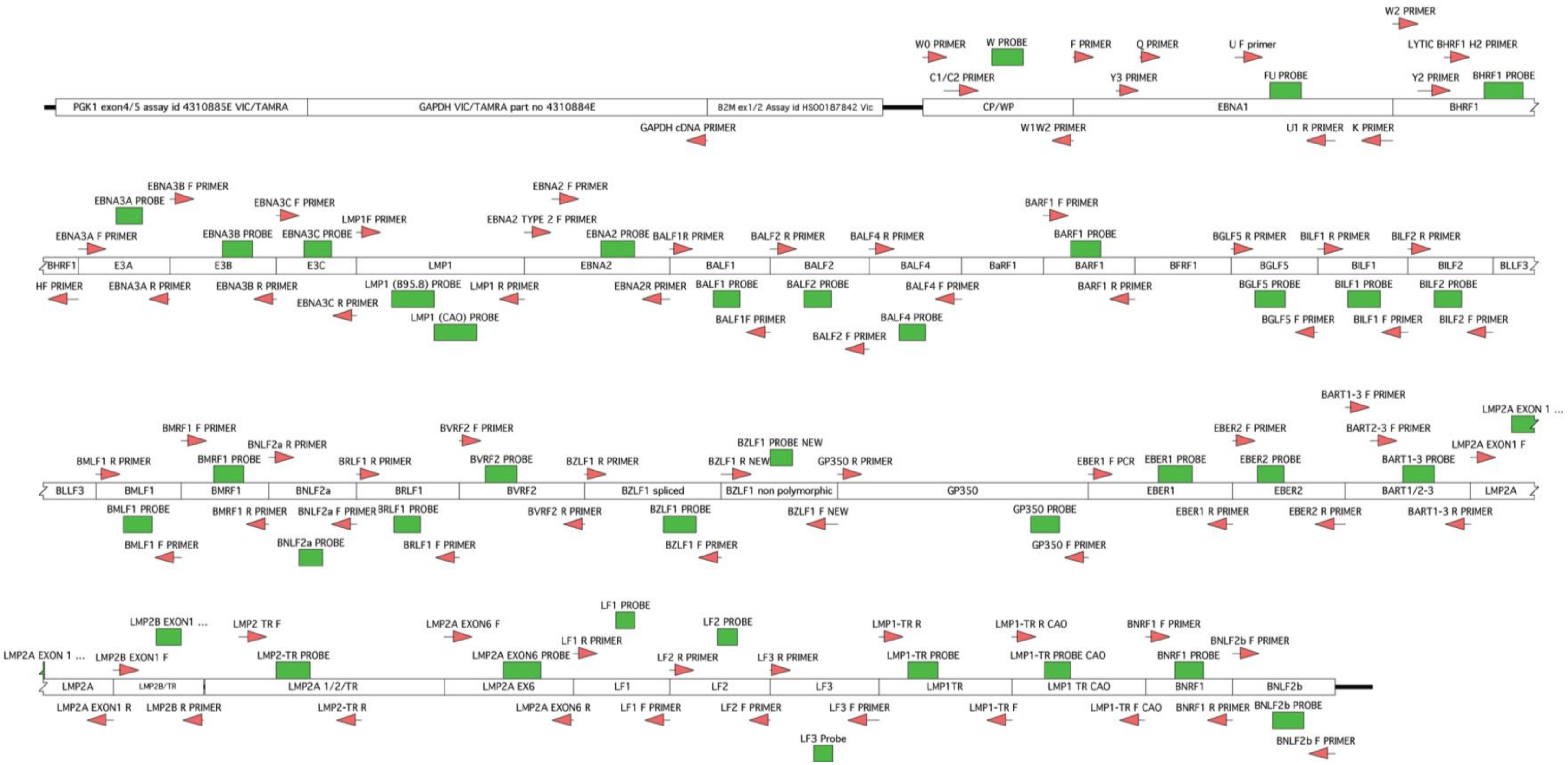

Supplementary Fig. S1. Schematic of the AQ EBV plasmid standard. A 4951 base pair DNA sequence was designed representing 45 contiguous latent and lytic EBV amplicons and 3 cellular amplicons. This sequence was commercially synthesized and obtained cloned into a pUC57 vector. The resulting plasmid (AQ-plasmid) was used at 10 fold dilutions containing 10^{-1} - 10^{5} copies/µl to generate standard curves for QPCR. The red arrows indicate the positions of the sequences complementary to the forward and reverse primers for the PCR assays, and the green rectangles indicate the positions of the TaqMan probe sequences. The sequences of primers and probes are listed in Table S1.

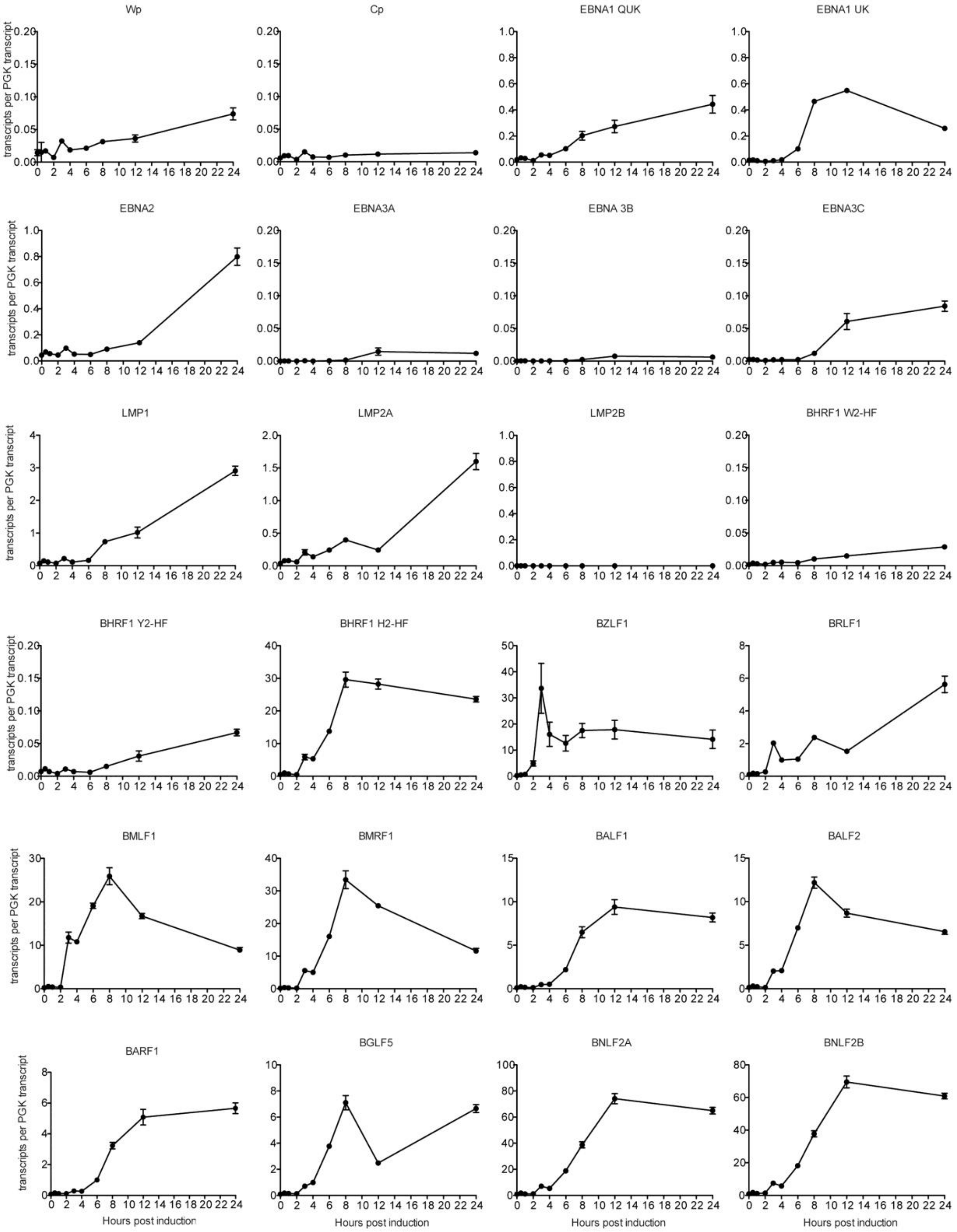
Wp
Cp
EBNA1 QUK
EBNA1 UK
EBNA2
EBNA3A
EBNA 3B
EBNA3C
LMP1
LMP2A
LMP2B
BHRF1 W2-HF
BHRF1 Y2-HF
BHRF1 H2-HF
BZLF1
BRLF1
BMLF1
BMRF1
BALF1
BALF2
BARF1
BGLF5
BNLF2A
BNLF2B
transcripts per PGK transcript
Hours post induction

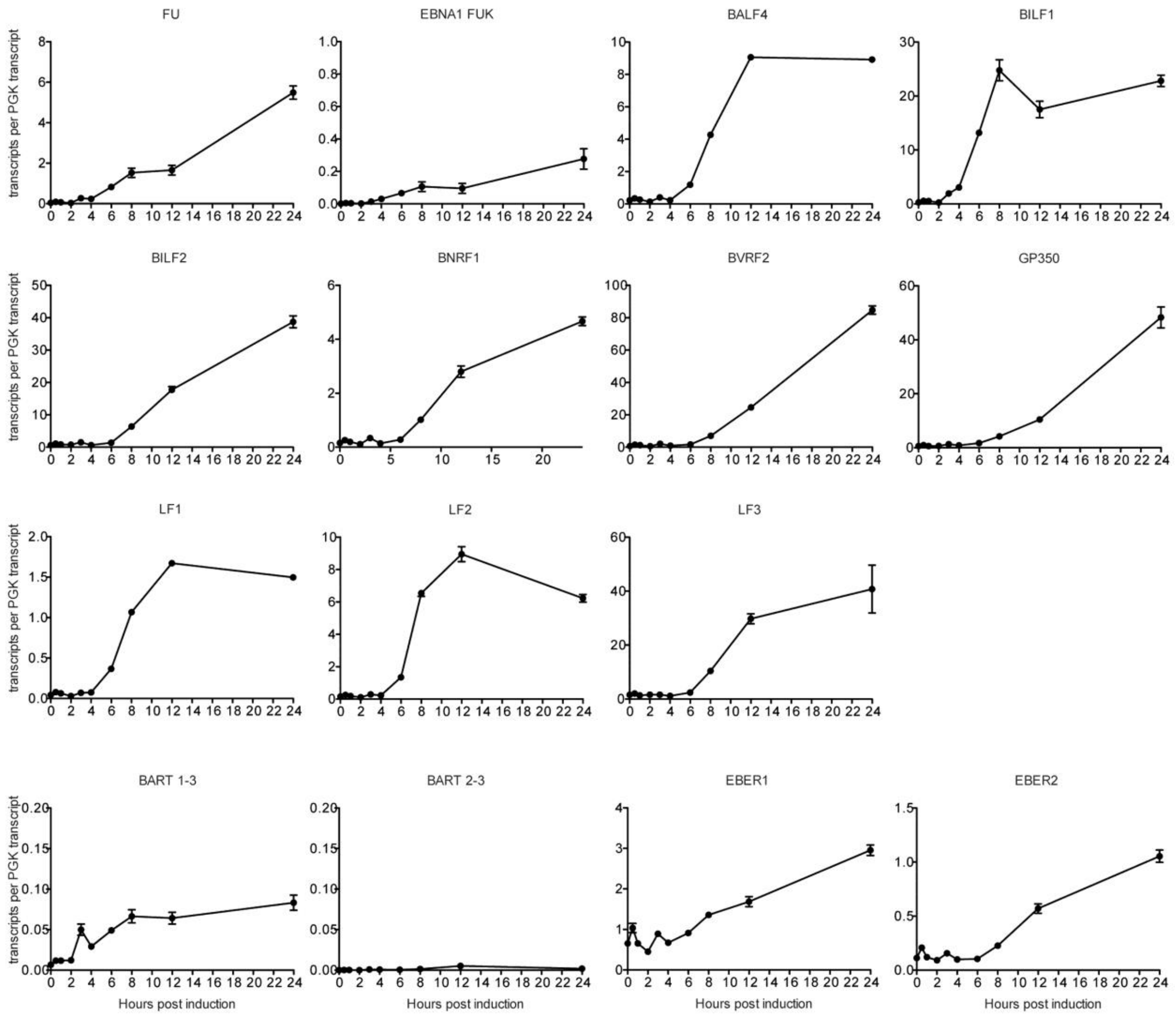

Supplementary Fig. S2. Kinetics of EBV transcript changes during induction of lytic cycle. Quantitation of 39 EBV transcripts in Akata BL cells over a 24 hour period following induction of lytic cycle by anti-IgG treatment. Absolute numbers of each transcript in each sample was determined from standard curves obtained with the AQ plasmid. The data are plotted as the number of EBV transcripts divided by the number of cellular PGK transcripts in each sample. Error bars represent the SEM of triplicate assays.

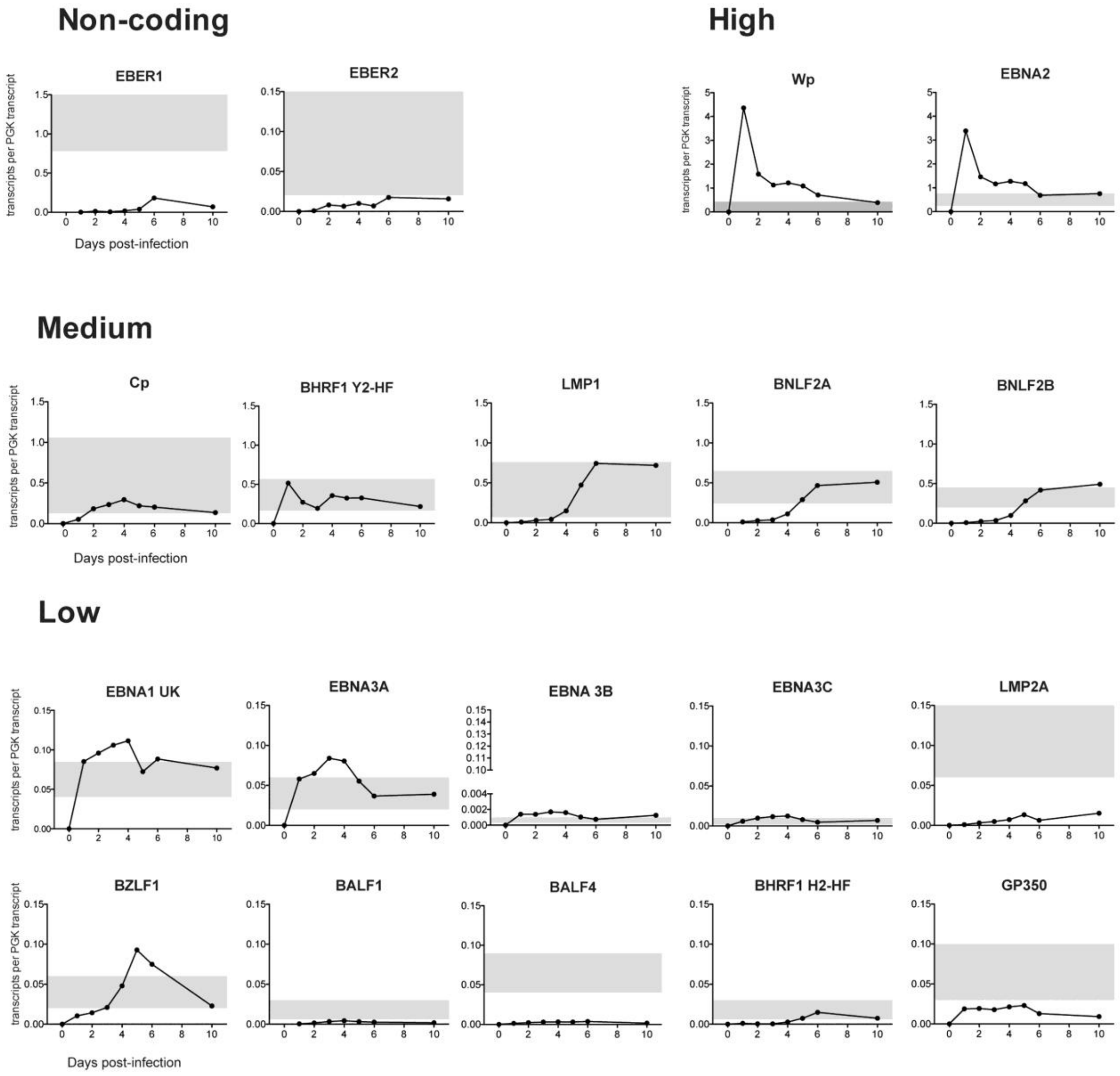

Supplementary Fig. S3. Kinetics of appearance of EBV transcripts during infection of normal B cells. Selected examples of EBV transcripts expressed at high, medium or low copy numbers in the first 10 days following infection of primary B cells with B95.8-derived 2089 recombinant EBV. Absolute numbers of each transcript in each sample was determined from standard curves obtained with the AQ plasmid. The data are plotted as the number of EBV transcripts divided by the number of cellular PGK transcripts in each sample. Shading represents the range of values obtained from 9 established LCLs.

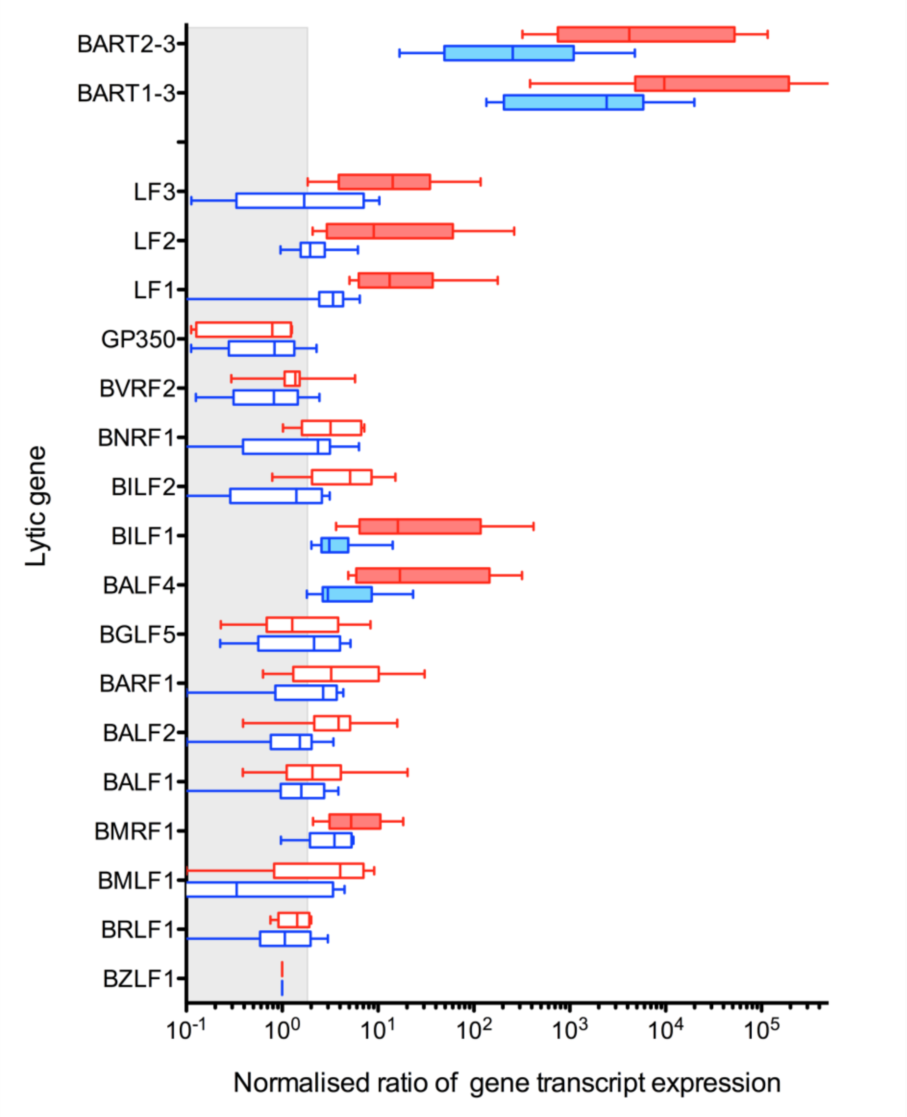

Supplementary Fig. S4. Expression of lytic genes in Lat I and Lat III lines. The boxplots illustrate gene expression data from 8 Lat I BL lines (red) and 10 Lat III lines (5 BL and 5 LCL; blue). For each illustrated gene in the two groups, the ratio of the number of target gene transcripts to the number of BZLF1 transcripts is shown normalized to the corresponding ratio from 100% lytic Akata-BL cells. Genes shown in solid shading are more highly expressed than would be expected from the levels of BZLF1 expression.
