# Supplementary material for: Unexpected patterns of Epstein–Barr virus transcription revealed by a High throughput PCR array for absolute quantification of viral mRNA
Source: Virology. 2015 Jan 1;474:117–30. doi: 10.1016/j.virol.2014.10.030 (PMC4266535; doi:10.1016/j.virol.2014.10.030)
Supplement: Supplementary file 2 — Supplementary material [file mmc2.doc]

Table S1. Details of primer/probe combinations to detect EBV transcripts

| Assay | Primer/Probe | EBV genome  coordinates * | Sequence | 20x conc. |
| --- | --- | --- | --- | --- |
| Wp | W0 | 14391–14410 | CGCCAGGAGTCCACACAAAT | 2 µM |
|  | W1W2 | 14709–14701/  14619–14612 | GAGGGGACCCTCTGGCC | 2 µM |
| Cp | C1C2 | 11467–11479/  11626–11639 | AATCATCTAAACCGACTGAAGAAACAG | 20 µM |
|  | W1W2 | 14709–14701/  14619–14612 | GAGGGGACCCTCTGGCC | 20 µM |
| Wp/Cp | W probe | 14564–14588 | ACCGAAGTGAAGGCCCTGGACCAAC | 4 µM |
|  |  |  |  |  |
| EBNA1 | Q | 62440–62456 | GTGCGCTACCGGATGGC | 20 µM |
|  | Y3 | 48422–48440 | TGCCTGAACCTGTGGTTGG | 20 µM |
|  | F primer | 50099-50115 | GGGTGAGGCCACGCTTT | 20 µM |
|  | U | 55247-55269 | CTGCAGCCCAGAGAGTAGTCTCA | 20 µM |
|  | U1 | 55326-55304 | CAGGTCTACTGGCGGTCTATGAT | 20 µM |
|  | K | 107952–107941/  67649–67636 | CATGATTCACACTTAAAGGAGACGG | 20 µM |
|  | U probe | 67563–67587 | TCCTCTGGAGCCTGACCTGTGATCG | 4 µM |
|  |  |  |  |  |
| EBNA2 | EBNA2 Type1 F | 35702-35711  36098-36109/ | GCTTAGCCAGTAACCCAGCACT | 6 µM |
|  | EBNA2 Type2 F |  | GCTTAGCCAGTAACTCAGCGCT | 6 µM |
|  | EBNA2 R | 36181-36160 | TGCTTAGAAGGTTGTTGGCATG | 6 µM |
|  | EBNA2 probe | 36127-36153 | CCCAACCACAGGTTCAGGCAAAACTTT | 4 µM |
|  |  |  |  |  |
| EBNA3A | EBNA3A F | 81944-81929 | CCCCTTAACTCAACCCATTAACC | 6 µM |
|  | EBNA3A R | 82313-82335 | CGGCCCCTCCATTGGT | 6 µM |
|  | EBNA3A probe | 82343-82363 | ACCCGCAGCCCATTTCCTCCA | 4 µM |
|  |  |  |  |  |
| EBNA3B | EBNA3B F | 83265-83281 | TGCCGCTGCAAGAGAGG | 20 µM |
|  | EBNA3B R | 83510-83500/  83421-83413 | AGGTCCGATTGCAACATGGA | 20 µM |
|  | EBNA3B probe | 83347-83374 | CCTAGATTTGTGGATGTGAACCCAACGC | 4 µM |
|  |  |  |  |  |
| EBNA3C | EBNA3C F | 88813-88831 | TACGCCCCATTCCAACAAG | 6 µM |
|  | EBNA3C R | 88876-88858 | CCCACGGCCATGCTATCTT | 6 µM |
|  | EBNA3C probe | 88835-88856 | CCCCCCTCCCCCTATGCCGTTA | 4 µM |
|  |  |  |  |  |
| BHRF1 | W2 | 14810-14830 | TGGTAAGCGGTTCACCTTCAG | 6 µM |
|  | Y2 | 35706-35680 | GAGGATGAAGACTAAGTCACAGGCTTA | 6 µM |
|  | H2 | 41596-41607/  42047-42055 | GGCTTACCTCGGTTCCCTCTT | 6 µM |
|  | HF | 42134-42111 | TCCCGTATACACAGGGCTAACAGT | 6 µM |
|  | Probe | 42067-42097 | TGCCAGATCTTGTAGAGCAAGATGGCCTATT | 4 µM |
|  |  |  |  |  |
| LMP1 | LMP1 F | 168644-168625 | AATTTGCACGGACAGGCATT | 6 µM |
|  | LMP1R | 168435-168454 | AAGGCCAAAAGCTGCCAGAT | 6 µM |
|  | LMP 1 probe (B95) | 168951-168965/  169042-169060 | TCCAGATACCTAAGACAAGTAAGCACCCGAAGAT | 4 µM |
|  | LMP1 probe (Cao) |  | TCCAGAGACCTAAGACAAGTAAGCAGCCAAAGAT | 4 µM |
|  |  |  |  |  |
| LMP1-TR | LMP1-TR F (B95) | 169013-168994 | CCCCTCTCAAGGTCGTGTTC | 6 µM |
|  | LMP1-TR F (Cao) |  | GCGTCTCAAGGTCGCGTTC | 6 µM |
|  | LMP1-TR R (B95) | 169099-169080 | CGTAGCCGCCCTACATAAGC | 6 µM |
|  | LMP1-TR R (Cao) |  | TCGTAGGCGGCCTACATAACC | 6 µM |
|  | LMP1-TR probe (B95) | 169017-169040 | CCTCAGGGCAGTGTGTCAGGAGCA | 4 µM |
|  | LMP1-TR probe (Cao) |  | TGCTCCTGCCACACTACCCTGACCA | 4 µM |
|  |  |  |  |  |
| LMP2 (total) | LMP2 exon6 F | 1027-1049 | GGTTCTCCTGATTTGCTCTTCGT | 6 µM |
|  | LMP2 exon6 R | 1129-1113 | CGCGGAGGCTAGCAACA | 6 µM |
|  | LMP2 exon6 probe | 1074-1103 | TCCTTCTGGCACGACTGTTCCTATATGCTC | 4 µM |
|  |  |  |  |  |
| LMP2A | LMP2A exon1 F | 166109-166129 | TCCCTAGAAATGGTGCCAATG | 6 µM |
|  | LMP2A exon1 R | 166215-166195 | GAAGAGCCAGAAGCAGATGGA | 6 µM |
|  | LMP2A probe | 166142-166160 | CCTAGCCCCGGCGGGGATC | 4 µM |
|  |  |  |  |  |
| LMP2B | LMP2B exon1F | 169365-169385 | GTAATCTGCACAAAGAGGCGC | 6 µM |
|  | LMP2B exon1R | 169436-169421 | AAAGCACGGCCTCCCG | 6 µM |
|  | LMP2B probe | 169399-169418 | TGCCGCCAACGACCTCCCAA | 4 µM |
|  |  |  |  |  |
| LMP2-TR | LMP2-TR F | 28-50 | ACTTTTCTTCTTGCCCGTTCTCT | 6 µM |
|  | LMP2-TR R | 114-133 | GAAACACGAGGCGGCAATAG | 6 µM |
|  | LMP2-TR probe | 66-92 | CAGTATGCCTGCCTGTAATTGTTGCGC | 4 µM |
|  |  |  |  |  |
| BZLF1 | BZLF1 F | 90859-90835 | CCCAAACTCGACTTCTGAAGATGTA | custom |
|  | BZLF1 R | 90767-90791 | TGATAGACTCTGGTAGCTTGGTCAA | assay |
|  | BZLF1 probe | 90803-90820 | CCCATACCAGGTGCCTTT |  |
|  |  |  |  |  |
| BRLF1 | BRLF1 F | 92662-92644 | TTGGGCCATTCTCCGAAAC | 6 µM |
|  | BRLF1 R | 92581-92590 | TATAGGGCACGCGATGGAA | 6 µM |
|  | BRLF1 probe | 92611-92631 | AGACGGGCTGAGAATGCCGGC | 4 µM |
|  |  |  |  |  |
| BMLF1 | BMLF1 F | 71978-71958 | CCCGAACTAGCAGCATTTCCT | 6 µM |
|  | BMLF1 R | 71805-71824 | GACCGCTTCGAGTTCCAGAA | 6 µM |
|  | BMLF1 probe | 71941-71955 | AACGAGGATCCCGCAGAGAGCCA | 4 µM |
|  |  |  |  |  |
| BALF1 | BALF1 F | 164694-169676 | GGGCAAAGACACGCACGTA | 6 µM |
|  | BALF1 R | 164615-164633 | GCCGCGACCAGTAGTCGTA | 6 µM |
|  | BALF1 probe | 164650-164670 | CATCATCAGCGTCCTGCGCGC | 4 µM |
|  |  |  |  |  |
| BALF2 | BALF2F | 164131-164113 | CGGGCTTCAGCATCAATGT | 6 µM |
|  | BALF2R | 164053-164074 | TGATAGGAGGTAGCGCGTAGGA | 6 µM |
|  | BALF2 probe | 164080-164101 | ACAGGAGGCCCGACCCCAACTG | 4 µM |
|  |  |  |  |  |
| BARF1 | BARF1 F | 165586-165606 | GGGAGCCTCTCTGTTGCTGTT | 6 µM |
|  | BARF1 R | 165658-165639 | TTTTCCCAACGCAGGTCACT | 6 µM |
|  | BARF1 probe | 165608-165631 | ACCTGTCACTTCCCAAGCCCTGGC | 4 µM |
| BGLF5 | BGLF5F | 108930-108913 | GCAAGCCCGGGAGAGACT | 6 µM |
|  | BGFL5R | 108862-108879 | GAGGCGACCGTTTTCGAA | 6 µM |
|  | BGLF5 probe | 108881-108904 | CGGGTGAACATTGTGACGGCCTTC | 4 µM |
|  |  |  |  |  |
| BHRF1 | H2 | 41596-41606/  42047-42056 | GGCTTACCTCGGTTCCCTCTTA | 6 µM |
|  | HF | 42111-42134 | TCCCGTATACACAGGGCTAACAGT | 6 µM |
|  | BHRF1 probe | 42067-42097 | AATAGGCCATCTTGCTCTACAAGATCTGGCA | 4 µM |
|  |  |  |  |  |
| BMRF1 | BMRF1F | 68078-68098 | GAGGAACGAGCAGATGATTGG | 6 µM |
|  | BMRF1R | 68147-68130 | TGCCCACTTCTGCAACGA | 6 µM |
|  | BMRF1 probe | 68104-68127 | TGCTGTTGATGCCCAAGACGGCTT | 4 µM |
|  |  |  |  |  |
| BNLF2a | BNLF2a F | 167015-166996 | TGGAGCGTGCTTTGCTAGAG | 6 µM |
|  | BNLF2a R | 166946-166966 | GGCCTGGTCTCCGTAGAAGAG | 6 µM |
|  | BNLF2a probe | 166970-166988 | CCTCTGCCTGCGGCCTGCC | 4 µM |
|  |  |  |  |  |
| BNLF2b | BNLF2b F | 166828-166808 | GGAGTTTCCCCCGATTCAAG | 6 µM |
|  | BNLF2b R | 166748-166769 | AAAGGTCAAAGAACAAGGCCAA | 6 µM |
|  | BNLF2b probe | 166780-166804 | TCTGAAGGAACGGCGGAGAGTAGCG | 4 µM |
|  |  |  |  |  |
| BALF4 | BALF4F | 157766-157784 | CCTGGCCCTTCGTGTAACG | custom |
|  | BALF4R | 157835-157813 | GAAGAGCAGGTGAACAAGACCAT | assay |
|  | BALF4 probe | 157792-157807 | ACGGCCTCGTACTTCT |  |
|  |  |  |  |  |
| BILF1 | BILF1 F | 152353-152333 | TGCCTTTTGACCCAGAACATG | 6 µM |
|  | BILF1 R | 152282-152303 | CAACGCCATACCCAAGTGAGT | 6 µM |
|  | BILF1 probe | 152306-152331 | TACGGAGCACATCAGGCCCAAGAACA | 4 µM |
|  |  |  |  |  |
| BILF2 | BILF2F | 138118-138098 | GCAGCGTTCCTAGCAATGAGT | 6 µM |
|  | BILF2R | 138051-138069 | TCCATCCCCGGGAGTGTAT | 6 µM |
|  | BILF2 probe | 138072-138093 | CTCTCGGATCGAGTTGGGCCGG | 4 µM |
|  |  |  |  |  |
| BNRF1 | BNRF1 F | 3463-3482 | GGAGTTTCCCCCGATTCAAG | 6 µM |
|  | BNRF1 R | 3531-3512 | AAAGGTCAAAGAACAAGGCCAA | 6 µM |
|  | BNRF1 probe | 3486-3508 | AGGGCGCAAGTTCTCCGGTACCC | 4 µM |
|  |  |  |  |  |
| BVRF2 | BVRF2 F | 136089-136108 | CCACGGCAGTCTACGGTACA | 6 µM |
|  | BVRF2 R | 136188-136173 | GCGGCATTGGCGTCAT | 6 µM |
|  | BVRF2 probe | 136110-136134 | ACCTTGCGTGGGTCCTGAAGCACTT | 4 µM |
|  |  |  |  |  |
| BLLF1/  GP350 | GP350 F | 77316-77297 | AGAATCTGGGCTGGGACGTT | 6 µM |
|  | GP350 R | 77478-77496 | ACATGGAGCCCGGACAAGT | 6 µM |
|  | GP350 probe | 77451-77473 | AGCCCACCACAGATTACGGCGGT | 4 µM |
|  |  |  |  |  |
| FU | F1 | 50099-50115 | GGGTGAGGCCACGCTTT | 10 µM |
|  | U1 R | 55326-55304 | CAGGTCTACTGGCGGTCTATGAT | 10 µM |
|  | U probe | 55275-55299 | TCCTCTGGAGCCTGACCTGTGATCG | 4 µM |
|  |  |  |  |  |
| LF1 | LF1 F | 150962-150943 | GACTGACTCAGGGCCACATC | custom |
|  | LF1 R | 151000-151019 | AGAAAGCGGGCCCATGAAGG | assay |
|  | LF1 probe | 150977-150991 | ACGCCGCCTCGCCAG |  |
|  |  |  |  |  |
| LF2 | LF2 F | 149269-149286 | CCGGACCGTCAGCTTGAG | custom |
|  | LF2 R | 149335-149314 | CAACCCGGTCTTCTACGTCTAC | assay |
|  | LF2 probe | 149295-149310 | CCGGCTTCCACTCCTG |  |
|  |  |  |  |  |
| LF3 | LF3 F | 140769-140753 | AGGGCTGGGTCCTGAGA | custom |
|  | LF3 R | 140683-140707 | ACACGTGATGTAAGTTTAGCCAGTT | assay |
|  | LF3 probe | 140720-140734 | GACTTTCGGGGCATT |  |
|  |  |  |  |  |
| EBER1 | EBER1 F | 6654-6674 | TGCTAGGGAGGAGACGTGTGT | 6 µM |
|  | EBER1 R | 6768-6749 | TGACCGAAGACGGCAGAAAG | 6 µM |
|  | EBER1 probe | 6710-6736 | AGACAACCACAGACACCGTCCTCACCA | 4 µM |
|  |  |  |  |  |
| EBER2 | EBER2 F | 6994-7012 | AACGCTCAGTGCGGTGCTA | 6 µM |
|  | EBER2 R | 7083-7060 | GAATCCTGACTTGCAAATGCTCTA | 6 µM |
|  | EBER2 probe | 7014-7034 | CGACCCGAGGTCAAGTCCCGG | 4 µM |
|  |  |  |  |  |
| BART1-3 | BART1-3 F | 149581-149587 | CTCTTCATGTGAGGTCCGGC | 6 µM |
|  | BART3 R | 149645-149626 | TGTGTCCGGTAAACGCCATA | 6 µM |
|  | BART3 probe | 149592-149616 | CCACGGAGACTCGGACGTAGCCCTT | 4 µM |
|  |  |  |  |  |
| BART2-3 | BART2-3 F | 146318-146334 | TCCACTTTGTGTTACAGGTCCG | 6 µM |
|  | BART3 R | 149645-149626 | TGTGTCCGGTAAACGCCATA | 6 µM |
|  | BART3 probe | 149592-149616 | CCACGGAGACTCGGACGTAGCCCTT | 4 µM |
|  |  |  |  |  |
| GAPDH | Spans exons |  | Assay ID hs99999905.m1 FAM/TAMRA  Applied Biosystems |  |
| PGK1 | Exon 4/5 assay |  | Assay ID hs99999906.m1 FAM/TAMRA  Applied Biosystems |  |
| B2M | Spans exons |  | Assay ID hs00187842 FAM/TAMRA  Applied Biosystems |  |

* EBV coordinates refer to the B95-8 wild type genome (accession number NC_007605.1)

Table S2. Cellular PGK1 and viral EBNA1 transcript levels per cell

| Cell line | Transcripts/cell | |
| --- | --- | --- |
|  | Cellular PGK | EBV EBNA1 (UK) |
| LCL-A | 120.4 | 8.2 |
| LCL-B | 160.4 | 7.8 |
| LCL-C | 146.7 | 5.6 |
| Kem-BL | 133.2 | 8.7 |
| Mutu-BL | 207.5 | 4.8 |
| mean ± s.d. | 154 ± 30 | 7.0 ± 1.5 |

**Table S3.** Protein and mRNA copy number data for selected cellular genes extracted from Schwanhaüsser et al, 2011, Nature 473: 337 342

**
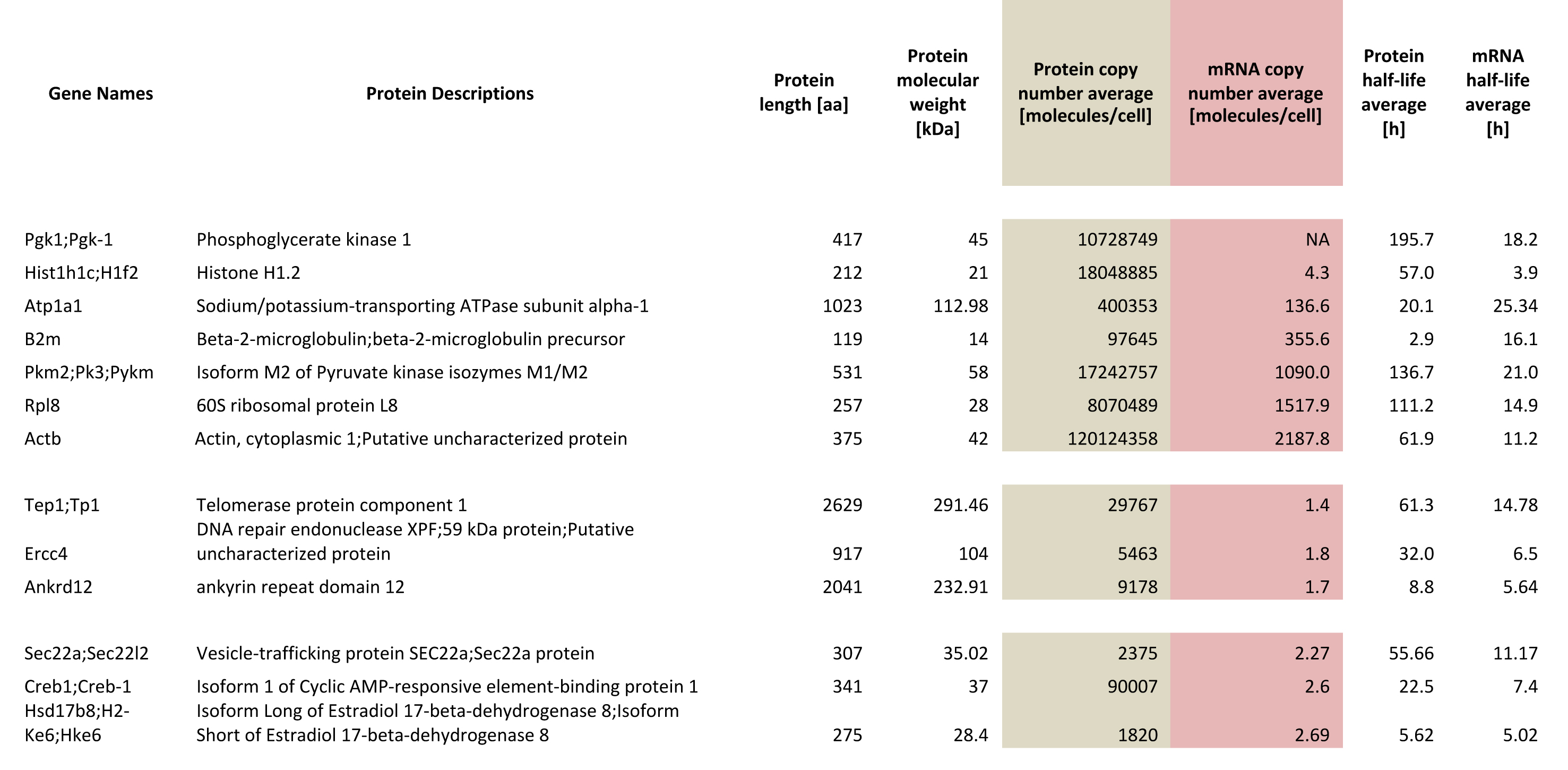
**

**Table S4.** Estimation of EBV transcripts per cell in lytic cycle and latent infection

| **Transcript** | **Lytic^†^** |  | **Lat I (n=8) ^‡^** | |  | **Wp (n=5)** | |  | **Lat III (n=15) ^§^** | |
| --- | --- | --- | --- | --- | --- | --- | --- | --- | --- | --- |
|  |  |  | **mean** | **SD** |  | **mean** | **SD** |  | **mean** | **SD** |
| Wp | 237.3 |  | 0.2 | 0.2 |  | 1466.3 | 1514.4 |  | 36.0 | 25.2 |
| Cp | 196.0 |  | 0.3 | 0.5 |  | 1.7 | 3.5 |  | 67.8 | 71.2 |
| EBNA1-QUK | 7.2 |  | 9.4 | 12.0 |  | 4.1 | 7.8 |  | 0.4 | 0.3 |
| EBNA1 -UK | 83.0 |  | 4.8 | 5.9 |  | 11.7 | 10.2 |  | 8.7 | 4.7 |
| EBNA2 | 295.3 |  | 1.4 | 1.3 |  | 0.0 | 0.0 |  | 64.5 | 37.4 |
| EBNA3A | 1.7 |  | 1.2 | 1.8 |  | 18.2 | 17.0 |  | 6.7 | 5.9 |
| EBNA3B | 1.8 |  | 0.0 | 0.0 |  | 0.2 | 0.3 |  | 0.7 | 2.6 |
| EBNA3C | 24.0 |  | 0.4 | 0.4 |  | 3.8 | 5.2 |  | 1.0 | 1.0 |
| LMP1 | 281.2 |  | 3.8 | 5.9 |  | 0.2 | 0.3 |  | 75.0 | 74.8 |
| LMP2A | 829.3 |  | 2.5 | 2.8 |  | 7.1 | 11.2 |  | 14.3 | 8.8 |
| LMP2B | n.d. |  | 0.2 | 0.2 |  | 0.9 | 1.3 |  | 3.5 | 5.3 |
| BHRF1 W2-HF | 23.1 |  | 0.0 | 0.1 |  | 588.5 | 916.5 |  | 3.4 | 1.9 |
| BHRF1 Y2-HF | 112.3 |  | 0.5 | 0.5 |  | 0.0 | 0.1 |  | 51.2 | 33.9 |
| BHRF1 H2-HF | 3918.7 |  | 5.5 | 8.9 |  | 77.2 | 140.0 |  | 4.1 | 5.2 |
| BZLF1 | 4588.5 |  | 3.7 | 3.2 |  | 24.5 | 20.4 |  | 7.1 | 7.3 |
| BRLF1 | 2275.6 |  | 2.4 | 2.6 |  | 25.2 | 23.9 |  | 6.6 | 11.3 |
| BMLF1 | 951.8 |  | 3.7 | 4.7 |  | 3.8 | 5.6 |  | 1.7 | 2.5 |
| BMRF1 | 1240.9 |  | 5.6 | 5.7 |  | 27.7 | 23.4 |  | 6.1 | 5.0 |
| BALF1 | 1373.3 |  | 1.9 | 2.0 |  | 9.0 | 14.1 |  | 3.5 | 6.2 |
| BALF2 | 1381.8 |  | 3.2 | 3.7 |  | 11.6 | 14.1 |  | 3.2 | 4.6 |
| BARF1 | 698.6 |  | 1.4 | 1.4 |  | 6.0 | 9.8 |  | 2.6 | 4.4 |
| BGLF5 | 657.2 |  | 1.7 | 2.1 |  | 8.1 | 10.8 |  | 3.2 | 4.4 |
| BNLF2A | 6114.1 |  | 10.2 | 14.0 |  | 7.9 | 10.3 |  | 83.5 | 97.9 |
| BNLF2B | 2511.6 |  | 8.3 | 13.6 |  | 8.2 | 14.7 |  | 48.5 | 30.8 |
| FU | 85.8 |  | 2.7 | 2.9 |  | 11.8 | 24.0 |  | 12.9 | 23.6 |
| FUK | 5.3 |  | 0.1 | 0.1 |  | 0.5 | 0.9 |  | 0.3 | 0.2 |
| BALF4 | 1529.5 |  | 20.7 | 11.3 |  | 25.8 | 41.2 |  | 10.6 | 12.2 |
| BILF1 | 1486.2 |  | 20.6 | 13.6 |  | 47.6 | 76.4 |  | 8.5 | 8.7 |
| BILF2 | 1837.9 |  | 8.7 | 16.2 |  | 13.0 | 14.6 |  | 3.4 | 4.8 |
| BNRF1 | 1382.1 |  | 3.3 | 4.6 |  | 6.8 | 5.5 |  | 3.6 | 5.4 |
| BVRF2 | 3159.8 |  | 5.6 | 10.6 |  | 15.8 | 20.7 |  | 6.0 | 13.1 |
| GP350 | 7186.1 |  | 5.1 | 5.6 |  | 27.0 | 29.5 |  | 14.6 | 27.5 |
| LF1 | 657.8 |  | 5.6 | 3.0 |  | 11.3 | 22.7 |  | 2.9 | 3.2 |
| LF2 | 1144.2 |  | 8.0 | 5.1 |  | 7.3 | 10.4 |  | 4.1 | 3.6 |
| LF3 | 3942.5 |  | 32.2 | 34.8 |  | 44.5 | 72.5 |  | 24.2 | 34.9 |
| BART1-3 | 5.2 |  | 70.4 | 64.1 |  | 72.2 | 128.2 |  | 23.7 | 44.6 |
| BART2-3 | 0.3 |  | 1.3 | 1.4 |  | 1.7 | 3.6 |  | 0.3 | 0.7 |
| EBER1 | 3301.7 |  | 3462.2 | 4387.7 |  | 3765.6 | 3451.0 |  | 653.9 | 874.8 |
| EBER2 | 1899.8 |  | 403.3 | 506.6 |  | 489.0 | 324.1 |  | 54.6 | 82.4 |

**^†^** Data from Akata-BL cells induced into lytic cycle following anti-IgG crosslinking. Note that due to a sequence polymorphism, no data was obtained for the LMP2B assay.

**^‡^** Data from 8 Lat I BL cells lines described in Fig. 6B. Note that due to a LMP2B sequence polymorphism in Akata-BL cells, n=7 for the LMP2B assay.

**^§^** Data from 5 Lat III BL lines and 10 Lat III LCLs described in Fig. 6B. Due to the virus genome deletions in Raji BL, n=14 for EBNA3C, BALF1, BALF2, and BARF1. Due to the virus genome deletion in X50-7 LCL, n=14 for Cp. Due to virus genome deletion in B95.8-derived LCLs, n=10 for LF1, LF2, LF3, BART1-3, and BART2-3 assays.

n.d. not determined.
